# Determination of ecological statuses of streams in the Ceyhan River Basin using composition and ecological characteristics of diatoms

**DOI:** 10.1007/s11356-024-33518-0

**Published:** 2024-05-07

**Authors:** Ömer Lekesiz, Abuzer Çelekli, Mehmet Yavuzatmaca, Muzaffer Dügel

**Affiliations:** 1 Department of Biology, Faculty of Art and Science, Osmaniye Korkut Ata University, 80000 Osmaniye, Türkiye; 2 Department of Biology, Faculty of Art and Science, Gaziantep University, 27310 Gaziantep, Türkiye; 3 Department of Biology, Faculty of Arts and Science, Bolu Abant İzzet Baysal University, Gölköy, 14280 Bolu, Türkiye

**Keywords:** Biomonitoring, Water quality, Diatom indices, TIT, Lotic ecosystems

## Abstract

The present study aimed to evaluate the interactions between diatoms and ecological factors in various streams and to test the suitability of diatom indices to evaluate the ecological status of 44 streams in the Ceyhan River Basin during the spring and autumn periods of 2021 and the summer of 2022. Canonical correspondence analysis indicated the significant effects of electrical conductivity (EC), dissolved oxygen, biological oxygen demand (BOD_5_), total phosphorus (TP), and total nitrogen (TN) on the distribution of diatom species of streams in the Ceyhan River Basin. Of the streams, Aksu and Erkenez streams were associated with high EC, BOD_5_, and TP and characterized by pollution-tolerant species. Pollution-sensitive species showed close integration with Aksu Spring Brook, Gözpınar Creek, Göksun Creek, and Yeşilgöz Spring Brook, which related to a high dissolved oxygen gradient. Different eco-regional diatom indices displayed different scores, representing from bad to high ecological status in the Ceyhan River basin. Among the diatom indices, Trophic Index Turkey (TIT) proved to be the more suitable metric to assess the ecological status of streams. TIT indicated deterioration of water quality in Karasu (S06), Erkenez (S07) streams, and downstream areas of the Ceyhan River and the least distributed sampling stations in the basin. Results suggested that eco-regionally developed diatom indices, like TIT, are required to more accurately assess the ecological status of streams in the Mediterranean region. The study provides a fundamental assessment of the ecological status of streams in the Ceyhan River Basin using an appropriate diatom index before the Pazarcık-centered earthquake on February 6, 2023. Findings allow someone to assess the impact of the earthquake on diatom communities and ecological factors in the region in the future studies.

**Supplementary Information:**

The online version contains supplementary material available at 10.1007/s11356-024-33518-0.

## Introduction

Aquatic ecosystems around the world play crucial roles in the provision of fishing, drinking, irrigation, transportation, electricity, waste diffusion, and recreation (Dudgeon 2019; Sun et al. 2023). Increasing human activities (e.g., urbanization, land use, construction of dams, agricultural, and industrial activities) and human-induced global climate change have deteriorated the physical, chemical, and hydro-morphological conditions of aquatic ecosystems (Feio et al. 2021; Cheung et al. 2021). Global climate change can vary from one ecoregion to another (Gudmundsson et al. 2021; Tornés et al. 2022). This is particularly evident in the Mediterranean region, which is characterized by distinct climatic conditions and unique ecosystems (Malek et al. 2018). The availability of water in streams in the Mediterranean region is highly dependent on the precipitation patterns, temperature, and hydrological cycles directly influencing their health and functioning. The ongoing degradation of freshwater ecosystems has prompted the development of techniques and tools for biomonitoring to evaluate the degree of deterioration. Since the implementation of the European WFD-Water Framework Directive, bio-assessing the water quality of lotic ecosystems has been more crucial to achieve environmental sustainability (Hering et al. 2010; Birk et al. 2012; Kelly et al. 2014; Çelekli and Lekesiz 2020). Water scarcity, excessive water withdrawal from ecosystems for production, climate change, excessive evaporation, and lack of precipitation are some of the biggest obstacles for member states that have adopted WFD to reach good ecological status for aquatic bodies. In this context, solution-oriented water policies on water resources are extremely important for the holistic protection and management of water by all stakeholders. The concept of water safety is based on the protection of limited water resources in terms of quantity and quality and is closely related to watershed management for the sustainability of water (Falasco et al. 2021; Tan et al. 2021; Ochieng et al. 2022; Tornés et al. 2022). Biological monitoring is one of the most efficient, comprehensive, and economical ways to assess the water quality of aquatic ecosystems (Mangadze et al. 2019).

The bioassessment of streams using diatoms is a commonly used method in the literature (e.g., Çelekli et al. 2021; Costa and Schneck 2022; Viso and Blanco 2023). The answer to the question “Why are diatoms widely used in stream bioassessment?” lies in the following reasons: (i) they are sensitive to changes in environment; (ii) diatom species have specific preferences for water quality parameters such as nutrient levels, pH, temperature, and dissolved oxygen; (iii) there are more than 100,000 diatom taxa; (iv) they can be found in all aquatic ecosystems at any time of the year; and (v) they are well documented taxonomically along with their tolerance levels to environmental variables (Rott et al. 1999; Delgado and Pardo 2015; Lobo et al. 2015; Çelekli et al. 2022a). All these have enabled the development of diatom-based biotic indices in different ecological regions, such as the Eutrophication/Pollution Index (EPI-D)/Italy (Dell’Uomo et al. 2004), Trophic Index (TI)/Austria (Rott et al. 1999), Duero Diatom Index (DDI)/Spain (Álvarez-Blanco et al. 2013), Trophic Water Quality Index (TWQI)/Brazil (Lobo et al. 2015), and Trophic Index Turkey (TIT)/Turkey (Çelekli et al. 2019).

Biomonitoring involves the comparison of a sampling station with a reference site to assess water quality (European Commission 2003). It quantifies the extent to which situations deviate from references or least-disturbed conditions. Therefore, determination of reference sites has crucial importance but there are some challenges to detect these sites (Hering et al. 2010; Stubbington et al. 2018). In the process of determining the reference sites, it is necessary to establish diatom communities that accurately represent reference or least-disturbed conditions (Çelekli et al. 2022a, b). These communities can be utilized to define the pollution gradients from less to high, during the deviation of a site from the reference condition. Accordingly, when a lotic ecosystem is contaminated, pollution-sensitive diatom species decrease and/or disappear from the system or their numbers start to descend, when the pollution-tolerant organisms find suitable conditions to increase in variety and number. However, there are some challenges for detecting the potential reference conditions of streams with different typologies in the river basin catchment. Due to the reasons mentioned above, developed diatom indices have boundaries of ecological quality classes. Therefore, testing different indices and selecting the most suitable index for the studied region are crucial for accurately assessing the quality of aquatic environments. Unlike other diatom indices, TIT has ecological quality class limit values based on altitude, which is an important typological criterion. This situation is believed to contribute to the success of estimating the ecological status of sampling stations (Stubbington et al. 2018; Çelekli et al. 2022a, b).

Ceyhan River Basin includes various streams that display different hydromorphological characteristics in the Mediterranean region and are highly influenced by anthropogenic factors including agricultural practices, industrial activities, urbanization, and water resource management. Climate change is an important factor affecting the aquatic ecosystems in this region in the form of heatwaves and drought periods that have been stronger and longer in the recent years. Managing and mitigating the impacts of these anthropogenic activities is essential for the conservation and sustainable management of streams in the Ceyhan River Basin. Therefore, biological monitoring is one of the most appropriate ways to observe the ecological health and water quality of the streams in the Mediterranean region. Until now, there have been no comprehensive studies investigating the ecological characteristics of streams in the Ceyhan River Basin based on diatom assemblages. For this reason, the ecological assessment of various streams in the Ceyhan River basin was attempted for the first time in the literature, utilizing several eco-region diatom indices, land uses, and multivariate statistical methodologies. This is also the latest study providing information on the condition of streams in the province of Kahramanmaraş prior to Pazarcık-centered earthquake on February 6, 2023. The objectives of this study were to examine variations (i) in the relationships between diatom assemblages and ecological factors in various streams using multivariate approaches and (ii) in the suitability of various diatom indices, derived from different ecoregions, for evaluating the ecological status of these streams in the Ceyhan River Basin. The present study also included a combination of chemical and biological evaluations to predict the ecological status of the sampled streams in the Ceyhan River Basin. Determination of relationships between diatom species and environmental factors are more difficult in ecosystems. Therefore, multivariate techniques are used to quantify diatom-stressor interactions in complex environmental ecosystems (Delgado and Pardo 2015; Çelekli et al. 2022a, b). In the present study, multivariate approaches were used to elucidate relationships between diatom species and environmental factors in the Ceyhan River Basin.

## Material and methods

### The study area: Ceyhan River Basin

**Fig. 1:**
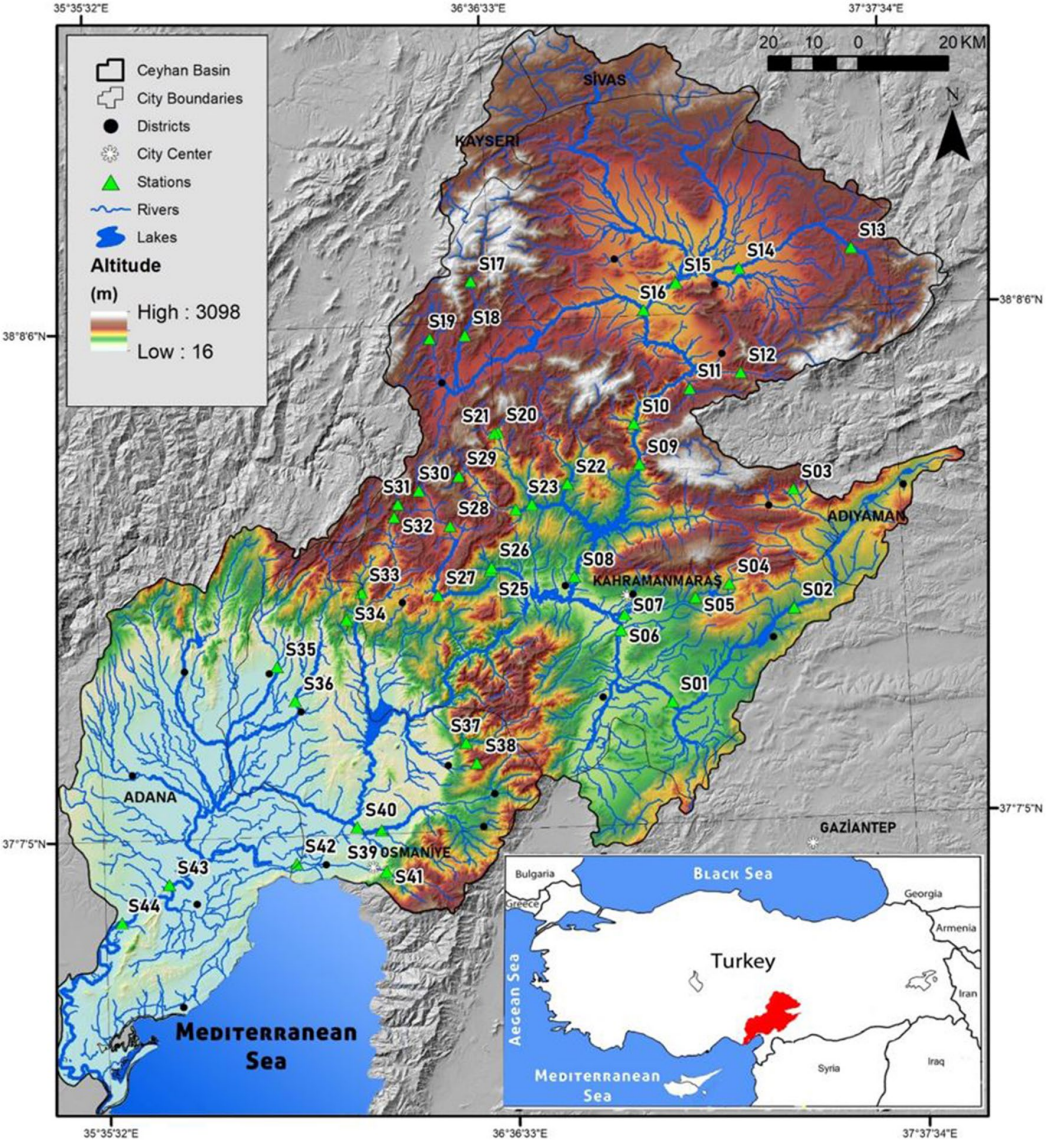
Distribution of sampled streams in the Ceyhan River Basin. Full names of streams coded from S01 to S44 are available in Table 1

The Ceyhan River Basin stretches from the center of the Central Anatolia Region to the Gulf of İskenderun in the semiarid Eastern Mediterranean region of Turkey (Fig. 1). The Ceyhan River, with about 510 km of length, begins in the mountains area around the Elbistan plain, merges with tributaries such as the Aksu and Göksu streams, and flows into the Mediterranean Sea. The basin is also home to various industrial facilities, including power plants, refineries, and manufacturing units. Industrial effluents and wastewater discharges may include pollutants such as heavy metals, chemicals, and organic compounds when they are not properly treated. Therefore, streams in the basin can become contaminated, directly affecting water quality and aquatic life. A significant part of the Ceyhan River Basin (32.4%) is used for agricultural purposes. The extensive use of fertilizers, pesticides, and herbicides for agricultural activities causes the leaking of these substances into streams. Excessive nutrient loading and chemical pollution cause the degradation of water quality, harm aquatic organisms, and disrupt the balance of stream ecosystems in the Ceyhan River Basin. The Mediterranean climate is dominant in the lower parts of the basin, while the continental climate is dominant in the middle and upper parts.

### Physicochemical analyses and sampling

During the field survey, 44 sampling stations (coded from S01 to S44) of streams in the Ceyhan River Basin were sampled during the spring and autumn periods of 2021 and the summer of 2022. Geographical Positioning System device (Garmin Etrex 10) was used to record coordinates and other geographical information about the stations. To draw the map (Fig. 1), 12.5 m of DEM (digital elevation model) data from Tandem-X, Alos-Palsar, Copernicus, Sentinel, and Landsat satellites were used for geographical and hydrological modeling. The hydrological network data (shapefile) and DEM data of our country obtained from the same satellites were transferred into a processable format using ArcGIS, ArcMAP 10.07, and GeoMapper 22.0 software. Table 1 lists the geographical information and full names of the sampled streams.

**Table 1:** Code, full name, and geographical data (coordinates and altitude) of each sampled streams

Code	Stream’s name	Longitude	Latitude	Altitude (m)
S01	Gökpınar Stream	37.36239	37.03063	531
S02	Aksu Stream1	37.53999	37.34684	746
S03	Aksu Spring Brook	37.77544	37.38601	1139
S04	Erkenez Stream1	37.59322	37.18652	919
S05	Erkenez Stream2	37.57324	37.10819	717
S06	Karasu Stream	37.50984	36.90867	453
S07	Erkenez Stream3	37.54063	36.92073	477
S08	Ceyhan River1	37.51545	36.92692	477
S09	Ceyhan River2	37.61983	36.79717	445
S10	Sarıgüzel Stream	37.92166	36.96325	803
S11	Nergele Stream	37.98722	37.10826	1163
S12	Gözpınar Creek	37.81868	36.96558	610
S13	Söğütlü Stream1	38.25441	37.53351	1350
S14	Söğütlü Stream2	38.22486	37.23786	1123
S15	Ceyhan River3	38.19979	37.08428	1114
S16	Göksun Stream1	38.14764	37.00002	1116
S17	Kavşut Stream	38.23841	36.58579	1570
S18	Çamdere Stream	38.11093	36.54157	1428
S19	Göksun Creek2	38.10745	36.44751	1430
S20	Yeşilgöz Spring Brook	37.91594	36.61908	1022
S21	Güredin Creek	37.91292	36.60929	1018
S22	Zeytin Stream	37.78034	36.77827	652
S23	Tekir Stream	37.76837	36.69721	655
S24	Kayaözü Stream	37.75815	36.63021	733
S25	Köprüağzı Stream1	37.63968	36.58646	500
S26	Karsulu Stream	37.64484	36.39985	505
S27	Köprüağzı Stream2	37.59671	36.44672	944
S28	Köprüağzı Stream3	37.73142	36.48229	1256
S29	Kumarlı Stream	37.83092	36.51509	1245
S30	Köprüağzı Stream4	37.81494	36.42205	1306
S31	Çiğsar Stream	37.77111	36.36455	1380
S32	Geben Stream	37.75105	36.34813	1280
S33	Keşiş Stream	37.60416	36.25824	743
S34	Kandırın Stream	37.55053	36.21918	687
S35	Sumbas Stream	37.45519	36.04103	97
S36	Savrun Stream	37.39124	36.08310	85
S37	Çatak Stream	37.29504	36.50844	477
S38	Karlıca Creek	37.25433	36.53388	1169
S39	Akçasu Stream1	37.12891	36.29320	99
S40	Akçasu Stream2	37.13415	36.22772	67
S41	Karaçay Stream	37.04428	36.29851	288
S42	Karasu Creek	37.06545	36.07368	38
S43	Cingöz Stream	37.03049	35.75386	29
S44	Ceyhan River	37.12891	36.29320	22

Physicochemical variables of water were measured before diatom sampling, in situ. A YSI professional plus oxygen-temperature multimeter was used to record the temperature (°C), dissolved oxygen (DO, mg/L), electrical conductivity (EC, μS/cm), pH, total dissolved solids (TDS, mg/L), and salinity (ppt) of the water. Then, water samples were taken from each sampled station in 500-mL polyethylene bottles and kept in cooler containers (+ 4 °C) until transfer to the Hydrobiology Laboratory of Gaziantep University for biological oxygen demand (BOD_5_) and chemical analyses.

The sampling of epilithic diatoms was made according to the standard methods of EN 13946 (European Committee for Standardization 2014). At least five stones in riffle regions of streams were randomly selected to represent the benthic diatom composition of the sampled station. The top surfaces of the taken stones were brushed with a soft-tipped brush and then washed with 100 mL of distilled water to allow the diatoms to dislodge from the stone surfaces. Once the samples reached sufficient density, they were fixed with a lugol-glycerol solution in the 250-mL plastic bottles.

### Laboratory analyses

The Hach Lange DR 5000 spectrophotometer was used to analyze total nitrogen (TN), and total phosphorus (TP) of water samples. These analyses were conducted after the Hach LT 200 Thermoreactor and Hach-Lange Bathtub tests. The Hach BOD Trak 2 device was used to measure biological oxygen demand (BOD_5_) (HACH 2005, 2010). These analyses were performed according to APHA (2012).

The potassium permanganate (KMnO_4_) was used to make permanent slides of epilithic diatom specimens according to the standard method of European Committee for Standardization (2014). Subsequently, permanent slides were examined under an Olympus BX53 light microscope with a DIC attachment and the cellSense Standard version CS-ST-V4.1 software on the basis of Windows OS (Win10) to count at least 450 diatom valves at × 1000 magnification. Taxonomic keys provided in Krammer (2000), Krammer (2002), Lange-Bertalot (2001), Bey and Ector (2013), and Lange-Bertalot et al. (2017) were used to identify diatom species. Algaebase (2023) was used for the taxonomic status of the species.

### Statistical analyses

The percentile analysis was used to determine the 25th, 50th, and 75th percentiles of environmental data. Before one-way ANOVA, homogeneity of variance and normality of data were tested by Levene’s test and Kolmogorov–Smirnov (*n* > 50) or Shapiro–Wilk (*n* < 50) tests, respectively. After requirements were met, one-way ANOVA with Duncan’s post hoc test were ran to test the differences in physicochemical data between/among sampling stations (SPSS version 15.0, USA).

Sampled stations were grouped according to their sampling seasons: spring, summer, and autumn. Then, analysis of similarity-ANOSIM was used to test whether there was a statistically significant difference among the diatom compositions of the sampling seasons (spring, summer, and autumn). If there was an important difference among the sampled seasons based on their diatom composition, an analysis of similarity percentage-SIMPER was used to determine which diatom species contributed to this dis/similarity among seasons. In the SIMPER analysis, species that displayed at least 1% contribution to the dis/similarity between groups (or sampling seasons) were considered. ANOSIM and SIMPER were performed with the aid of Community Analysis Package version 4.1.3 (Seaby and Henderson 2007).

The gradient lengths of the first two axes of the detrended correspondence analysis (> 4.0) indicated the suitability of the data for the application of unimodal CCA-canonical correspondence analysis. The used environmental variables were ln(x + 1) transformed to eliminate skewness and achieve a normal distribution except pH (ter Braak and Šmilauer 2002). The importance of environmental factors explaining the variation in species data was assessed using the forward selection of Monte Carlo permutation test with 999 unconstrained permutations. Multicollinearity among the environmental variables was tested with aid of Ecological Community Analysis II Software (Seaby and Henderson 2007). Accordingly, variables with an inflation factor larger than 10 indicating a possibility of multicollinearity were removed from the analyses. Conducted CCA analysis with selecting environmental variables showed statistically significant (*p* < 0.003) results. Then, we performed CCA to explore the relationships between five environmental variables (biological oxygen demand (BOD_5_), total phosphorus (TP), total nitrogen (TN), electrical conductivity (EC), and dissolved oxygen (DO)) and 211 diatom species in the Ceyhan River Basin with the aid of CANOCO 4.5 software. Optimum levels of diatom species for the environmental variables were estimated by a weighted average regression model in the CALIBRATION program (Juggins and ter Braak 1992). Diatom species (211 spp.) that appeared more than twice were used in the multivariate statistical analyses (Supplementary 1).

A non-parametric Spearman correlation analyses in SPSS (version 15.0, USA) was used to estimate statistically significant correlations among diatom-based indices and environmental variables.

### Bioassessment of sampling stations

Ecological status of sampling stations in Ceyhan River Basin was determined, using various ecoregional diatom indices, including European indices (the Eutrophication and/or Pollution Index-Diatom-EPI-D (Dell’Uomo et al. 2004), the Trophic Index-TI (Rott et al. 1999), the Pollution Sensitivity Index-IPS (Coste 1982), the Trophic Diatom Index-TDI (Kelly et al. 2008), Duero Diatom Index-DDI (Álvarez-Blanco et al. 2013), and Trophic index Turkey-TIT (Çelekli et al. 2019) and different ecoregions such as Diatom Ecological Quality Index (DEQI) in Mexico (Salinas-Camarillo et al. 2021), Trophic Water Quality Index (TWOI) in Brazil (Lobo et al. 2015), and an Australian index, Richmond River Diatom Index (RRDI) (Oeding and Taffs 2017)). The class-boundaries of the diatom indices mentioned above are given in the Supplementary 2. Regarding the development of diatom indices based on nutrients, IPS and DDI exhibit negative correlations with pollution levels in the environment, while the remaining indices show positive correlations.

## Results

### Physico-chemical characteristics of sampling stations

Results of a few physicochemical analyses are given in Table 2, and other environmental variables are represented in Supplementary 3. The high BOD_5_ values were determined at Erkenez (S07 = 225.67 mg/L) and Karasu (S06 = 209.44 mg/L) streams, whereas the low BOD_5_ values were found in Aksu Spring Brook (S03 = 3.81 mg/L) and Gözpınar Creek (S12 = 3.81 mg/L). Significantly low DO values were recorded in Karasu (S06 = 2.02 mg/L) and Erkenez (S07 = 3.39) streams when compared with Gözpınar Creek (S12 = 9.70 mg/L) and Yeşilgöz Spring Brook (S20 = 9.54 mg/L). The highest values of total phosphorus (TP) were recorded in the following orders: Erkenez Stream3 (S07 = 2052.67 mg/L), Karasu Stream (S06 = 1965 mg/L), Erkenez Stream1 (S04 = 325.67 mg/L), and Akçasu Stream (S40 = 212 mg/L). The uppermost electrical conductivity (EC) values were noted in the Karasu (S06 = 6163 µS/cm) and Erkenez (S07 = 2666 µS/cm) streams, but the lowest values were noted in the Aksu Spring Brook (S03), Gözpınar Creek (S12), Göksun Creek (S19), and Yeşilgöz Spring Brook (S20).

**Table 2:** Mean and standard deviations of some physico-chemical variables measured from the sampled streams (St.) in the Ceyhan River Basin during the three sampling periods

St	Temperature	EC	BOD_5_	DO	TP	TN
	°C	µS/cm	mg/L	mg/L	µg/L	mg/L
S01	19.1 ± 5.9^c−j^	534 ± 58^a^	10.79 ± 7.47^a^	4.89 ± 0.22^a−c^	71.6 ± 80.0^a^	5.93 ± 1.45^a^
S02	16.1 ± 4.1^a−ı^	484 ± 189^a^	12.92 ± 9.29^a^	7.11 ± 1.49^b−e^	91.0 ± 39.7^a^	2.49 ± 0.32^a^
S03	11.3 ± 6.6^a−d^	234 ± 45^a^	3.81 ± 2.92^a^	7.92 ± 2.00^c−e^	30.0 ± 23.5^a^	1.94 ± 0.74^a^
S04	14.5 ± 1.1^a−g^	427 ± 64^a^	7.09 ± 2.62^a^	6.73 ± 1.66^b−e^	325.6 ± 397.6^a^	1.75 ± 0.82^a^
S05	23.4 ± 0.0^ h−j^	552 ± 0^a^	15.61 ± 0.00^a^	6.90 ± 0.00^c−e^	89.0 ± 0.0^a^	2.77 ± 0.00^a^
S06	26.2 ± 1.7^j^	6163 ± 2036^c^	209.44 ± 122.63^b^	2.02 ± 1.02^a^	1965.0 ± 833.4^b^	19.08 ± 9.20^b^
S07	21.0 ± 3.7^e−j^	2666 ± 1163^b^	225.67 ± 173.46^b^	3.39 ± 2.54^a−b^	2052.6 ± 822.5^b^	32.01 ± 23.29^c^
S08	11.4 ± 1.6^a−d^	329 ± 57^a^	13.82 ± 12.34^a^	6.85 ± 2.09^c−e^	78.6 ± 31.6^a^	1.65 ± 0.28^a^
S09	13.2 ± 3.5^a−e^	280 ± 63^a^	5.44 ± 4.30^a^	8.63 ± 1.86^c−e^	78.6 ± 56.4^a^	1.50 ± 0.57^a^
S10	15.2 ± 0.0^a−i^	239 ± 0^a^	17.41 ± 0.00^a^	8.20 ± 0.00^c−e^	50.0 ± 0.0^a^	2.02 ± 000^a^
S11	13.9 ± 5.7^a−f^	323 ± 58^a^	7.61 ± 8.80^a^	6.71 ± 1.47^b−e^	104.3 ± 85.6^a^	1.41 ± 0.56^a^
S12	7.8 ± 1.4^a^	207 ± 67^a^	3.81 ± 3.87^a^	9.70 ± 2.43^c−e^	60.0 ± 49.1^a^	1.65 ± 0.49^a^
S13	14.9 ± 7.9^a−h^	259 ± 82 ^a^	5.08 ± 5.48^a^	7.80 ± 2.59^c−e^	61.0 ± 25.7^a^	1.15 ± 0.39^a^
S14	22.1 ± 8.9^f−j^	458 ± 201^a^	7.04 ± 8.05^a^	7.34 ± 2.60^b−e^	89.6 ± 62.9^a^	1.53 ± 0.53^a^
S15	12.1 ± 1.2^a−d^	379 ± 38^a^	25.19 ± 29.11^a^	6.75 ± 1.21^b−e^	147.5 ± 136.4^a^	2.31 ± 0.95^a^
S16	11.2 ± 3.7^a−d^	278. ± 19^a^	8.44 ± 8.37^a^	8.71 ± 1.77^c−e^	91.3 ± 51.0^a^	1.49 ± 0.73^a^
S17	11.5 ± 4.2^a−d^	238 ± 43^a^	5.36 ± 3.78^a^	7.34 ± 1.44^b−e^	53.0 ± 46.6^a^	2.04 ± 0.89^a^
S18	12.8 ± 3.7^a−e^	270 ± 47^a^	4.81 ± 4.65^a^	7.21 ± 1.41^b−e^	57.3 ± 48.3^a^	2.11 ± 0.66^a^
S19	8.4 ± 1.2^a^	215 ± 1^a^	7.92 ± 5.86^a^	8.48 ± 1.24^c−e^	60.3 ± 52.5^a^	1.85 ± 1.06^a^
S20	9.3 ± 2.5^a−b^	214 ± 32^a^	5.27 ± 4.35^a^	9.54 ± 2.19^e^	54.6 ± 50.0^a^	1.20 ± 0.67^a^
S21	11.7 ± 2.4^a−d^	232 ± 9^a^	7.57 ± 4.05^a^	8.33 ± 1.46^c−e^	77.0 ± 77.4^a^	1.85 ± 0.86^a^
S22	18.5 ± 8.3^c−j^	387 ± 104^a^	8.01 ± 6.99^a^	6.51 ± 2.79^b−e^	194.6 ± 196.6^a^	1.40 ± 0.33^a^
S23	13.3 ± 2.4^a−f^	310 ± 109^a^	8.51 ± 3.25^a^	7.88 ± 3.01^c−e^	83.0 ± 38.1^a^	1.98 ± 1.40.^a^
S24	10.7 ± 2.0^a−c^	261 ± 55^a^	5.55 ± 3.33^a^	9.09 ± 1.72^d−e^	86.3 ± 58.0^a^	1.31 ± 0.53^a^
S25	12.5 ± 1.2^a−e^	317 ± 27^a^	10.65 ± 10.1^a^	7.38 ± 1.44^b−e^	67.5 ± 65.7^a^	0.76 ± 0.74^a^
S26	14.8 ± 0.0^a−h^	344 ± 0^a^	31.88 ± 0.00^a^	8.30 ± 0.00^c−e^	41.0 ± 0.0^a^	0.64 ± 0.00^a^
S27	14.7 ± 4.3^a−g^	290 ± 52^a^	4.96 ± 2.88^a^	8.29 ± 2.18^c−e^	63.0 ± 51.2^a^	1.16 ± 0.71^a^
S28	18.8 ± 9.7^c−j^	270 ± 45^a^	4.95 ± 2.55^a^	6.23 ± 3.63^b−e^	44.0 ± 1.4^a^	1.08 ± 0.57^a^
S29	12.8 ± 7.0^a−e^	336 ± 119^a^	4.28 ± 1.34^a^	7.53 ± 2.07^c−e^	47.0 ± 20.4^a^	1.54 ± 1.42^a^
S30	10.7 ± 4.1^a−c^	212 ± 36^a^	2.72 ± 1.31^a^	7.60 ± 2.04^c−e^	67.0 ± 45.9^a^	1.25 ± 0.71^a^
S31	11.1 ± 4.1^a−d^	249 ± 39^a^	5.93 ± 2.97^a^	7.42 ± 1.60^c−e^	69.3 ± 39.0^a^	0.86 ± 0.56^a^
S32	11.1 ± 0.0^a−d^	364 ± 0^a^	15.65 ± 0.00^a^	8.50 ± 0.00^c−e^	15.0 ± 0.0^a^	1.30 ± 0.00^a^
S33	14.4 ± 2.8^a−g^	293 ± 11^a^	4.73 ± 1.92^a^	7.10 ± 2.53^b−e^	82.0 ± 44.8^a^	1.20 ± 0.51^a^
S34	14.2 ± 0.0^a−f^	469 ± 0^a^	13.39 ± 0.00^a^	7.90 ± 0.00^c−e^	20.0 ± 0.0^a^	0.45 ± 0.00^a^
S35	17.4 ± 2.8^b−ı^	317 ± 31^a^	6.48 ± 4.91^a^	7.32 ± 3.08^b−e^	104.0 ± 53.8^a^	2.29 ± 0.33^a^
S36	23.0 ± 8.1^ g−j^	489 ± 191^a^	5.48 ± 4.82^a^	5.85 ± 3.20^b−e^	197.3 ± 141.3^a^	4.74 ± 2.79^a^
S37	15.2 ± 3.8^a−i^	411 ± 50^a^	10.46 ± 11.35^a^	6.45 ± 2.14^b−e^	56.0 ± 18.3^a^	1.12 ± 0.45^a^
S38	13.7 ± 4.0^a−f^	158 ± 23^a^	3.83 ± 1.31^a^	6.90 ± 1.95^b−e^	66.3 ± 56.0^a^	0.95 ± 0.49^a^
S39	21.1 ± 1.9^e−j^	647 ± 81^a^	18.31 ± 23.46^a^	5.97 ± 2.41^b−e^	108.3 ± 62.5^a^	3.82 ± 2.18^b^
S40	23.6 ± 3.7^i−j^	685 ± 73^a^	19.54 ± 24.54^a^	5.02 ± 1.22^a−d^	212.0 ± 133.7^a^	5.74 ± 1.33^a^
S41	16.4 ± 4.82^a−ı^	460 ± 88^a^	14.22 ± 16.67^a^	6.90 ± 2.21^b−e^	70.3 ± 71.6^a^	1.09 ± 0.75^a^
S42	18.63 ± 3.3^c−j^	479 ± 64^a^	14.27 ± 16.87^a^	6.06 ± 2.27^b−e^	137.6 ± 4.1^a^	3.38 ± 0.59^a^
S43	19.7 ± 2.2^d−j^	922 ± 177^a^	21.33 ± 12.01^a^	6.45 ± 2.15^b−e^	214.0 ± 120.0^a^	14.20 ± 1.10^a^
S44	19.0 ± 0.8^c−j^	523 ± 39^a^	21.65 ± 15.95^a^	5.29 ± 1.31^a−d^	229.0 ± 89.0^a^	2.09 ± 0.43^a^

Different superscript letters in each column mean statistical differences at 0.05 level. Values with the same letters in the same column indicate that the values did not statistically different from each other according to the Duncan’s test

*EC* electrical conductivity, *DO* dissolved oxygen, *BOD*_*5*_ biological oxygen demand, *TN* total nitrogen, *TP* total phosphorus

### Diatom composition

**Fig. 2:**
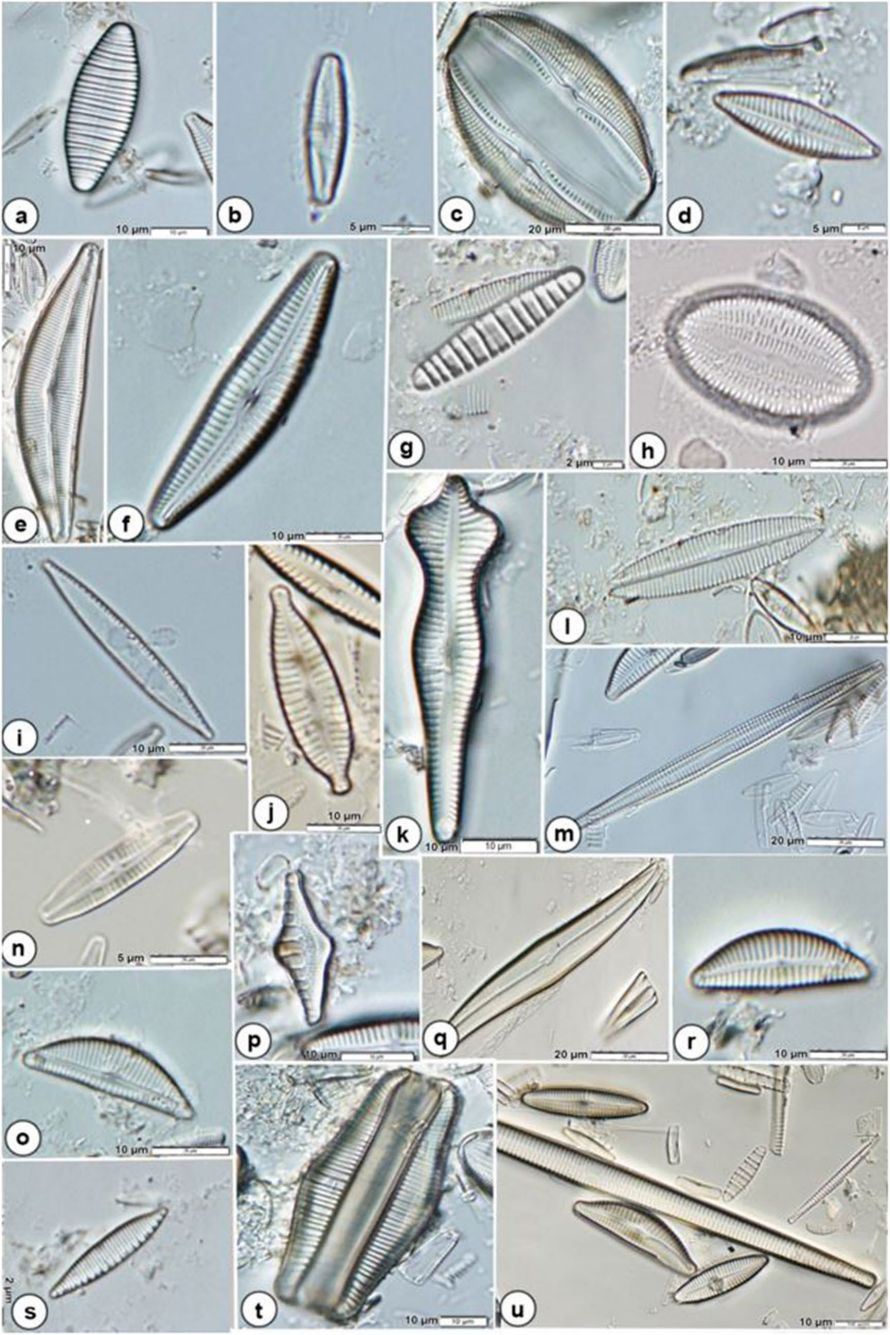
Commonly encountered diatom species in the present study. **a** *Diatoma vulgaris*, **b** *Achnanthidium pyrenaicum*, **c ***Amphora ovalis*, **d** *Navicula cryptotenella*, **e** *Cymbella compacta*, **f** *Cymbella excisiformis*, **g** *Diatoma moniliformis*, **h** *Cocconeis placentula*, **i** *Nitzschia palea*, **j** *Gomphonema parvulum*, **k** *Gomphonema acuminatum*, **l** *Navicula tripunctata*, **m** *Ulnaria acus*, **n** *Achnanthidium minutissimum*, **o** *Encyonema silesiacum*, **p** *Grunowia tabellaria*, **q** *Gyrosigma acuminatum*, **r** *Cymbella affinis*, **s** *Nitzschia amphibia*, **t** *Epithemia gibba*, and **u** *Ulnaria ulna*

During the studied periods, a total of 279 diatom taxa were identified in the sampled streams in the Ceyhan River Basin. Of the taxa, 211 (Supplementary 1) occurred at least twice and were used in the multivariate statistical analyses. Diatom species such as *Achnanthidium minutissimum*,* A. pyrenaicum*, *Amphora pediculus*, *Cocconeis placentula*, *Cymbella affinis*,* C. compacta*,* C. excisa*,* C. excisiformis*, *Diatoma moniliformis*, *Encyonema silesiacum*,* E. ventricosum*, *Gomphonema parvulum*, *Navicula cryptotenella*, *Nitzschia amphibia*,* N. palea*, *Odontidium mesodon*, *Ulnaria acus*, and *U. ulna* were commonly found in the present study (Fig. 2)*.*

There were significant differences in the diatom species composition among the sampling periods (spring, summer, and autumn), according to the ANOSIM results (*p* < 0.05). SIMPER analysis indicated a 28% within-group similarity in the spring. *Achnanthidium minutissimum*, *Diatoma moniliformis*, *Nitzschia palea*, and *Cymbella excisa* were primarily responsible for the 28% similarity in the spring season. In the autumn, *Cocconeis placentula*, *A. minutissimum*, *Amphora pediculus*, *Navicula capitatoradiata*, and *Cymbella affinis* showed great contributions to the 27% of in-group similarity. A 15% within-group similarity was found in the summer period, and *A. minutissimum*, *C. excisiformis*, *Achnanthidium gracillimum*, *Navicula tripunctata*, and *A. rivulare* were the most contributing species to this similarity.

SIMPER analysis also resulted in an 83% dissimilarity between the spring and autumn periods, with the important contributions of *A. minutissimum*, *C. placentula*, *D. moniliformis*, *Amphora pediculus*, *Nitzschia amphibia*, and *Nitzschia palea* species. The distinctness ratio between the spring and summer periods was 84%, and *A. minutissimum*, *D. moniliformis*, *Cymbella excisiformis*, *N. palea*, and *Achnanthidium gracillimum* species displayed important additions to this dissimilarity. *Cocconeis placentula*, *A. minutissimum*, *C. excisiformis*, *Amphora pediculus*, and *Nitzschia amphibia* were the species showing the highest percentage contributions to the 88% dissimilarity between autumn and summer periods.

### Diatom species and environmental relationships

**Fig. 3:**
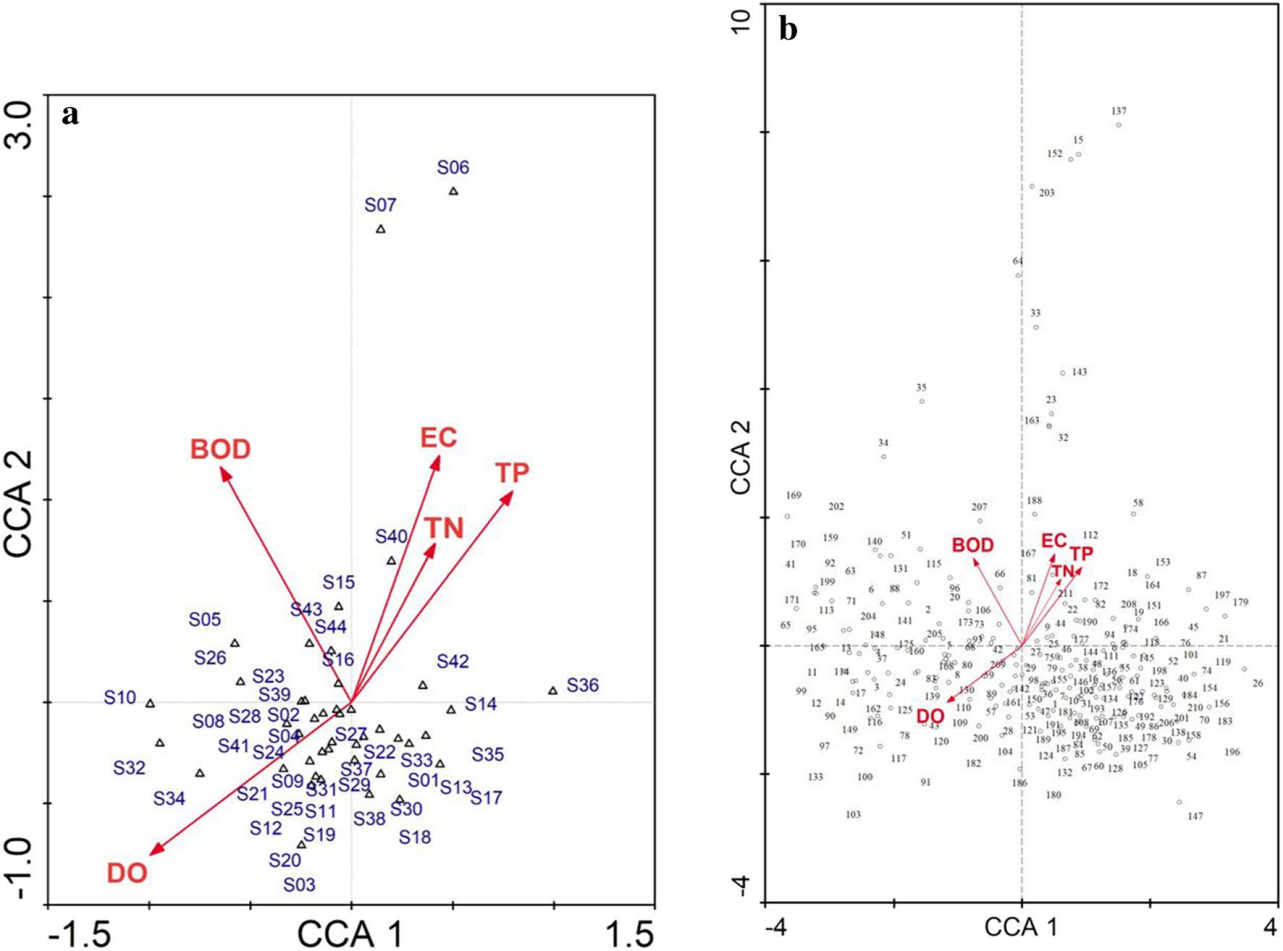
CCA diagram showing the relationships of (**a**) sampling stations (up-triangle) and (**b**) diatom species (circle) with environmental variables (arrow). Temp temperature, DO dissolved oxygen, BOD_5_ biological oxygen demand, TN total nitrogen, and TP total phosphorus. Full names of sampling stations and numbers representing the diatom species are given in Table 1 and Supplementary 1, respectively

Based on the first two axes of the CCA, 90.4% of the relationships between diatom species and environmental factors in the 44 sapling stations of various streams in the Ceyhan River Basin (Fig. 3a and b) was explained. Results of the CCA indicated significantly influential effects of biological oxygen demand (BOD_5_, *F* = 3.008, *p* = 0.001), total phosphorus (TP, *F* = 2.979, *p* = 0.001), electrical conductivity (EC, *F* = 2.914, *p* = 0.001), dissolved oxygen (DO, *F* = 2.815, *p* = 0.001), and total nitrogen (TN, *F* = 2.296, *p* = 0.002) on the distribution of diatom species composition in the present study.

Karasu (S06) and Erkenez (S07) streams were associated with high EC, TP, and TN in the Ceyhan River Basin (Fig. 3a) and characterized by pollution-tolerant species (Supplementary 1) such as *Navicula cincta* (coded 137), *Amphora copulata* (15), *Navicula recens* (152), *Surirella brebissoni* (203), and *Craticula ambigua* (33) (Fig. 3b). Pollution-sensitive species (Supplementary 1) such as *Neidiomorpha binodiformis* (160), *Meridion circulare* (133), *Denticula tenuis* (65), *Odontidium mesodon* (181), and *Cymbella affinis* (36) indicated close integrations with the stations relating to high dissolved oxygen gradients, as follows: Aksu Spring Brook (S03), Gözpınar Creek (S12), Göksun Creek (S19), Yeşilgöz Spring Brook (S20), Geben Stream (S32), and Kandırın Stream (S34).

**Fig. 4:**
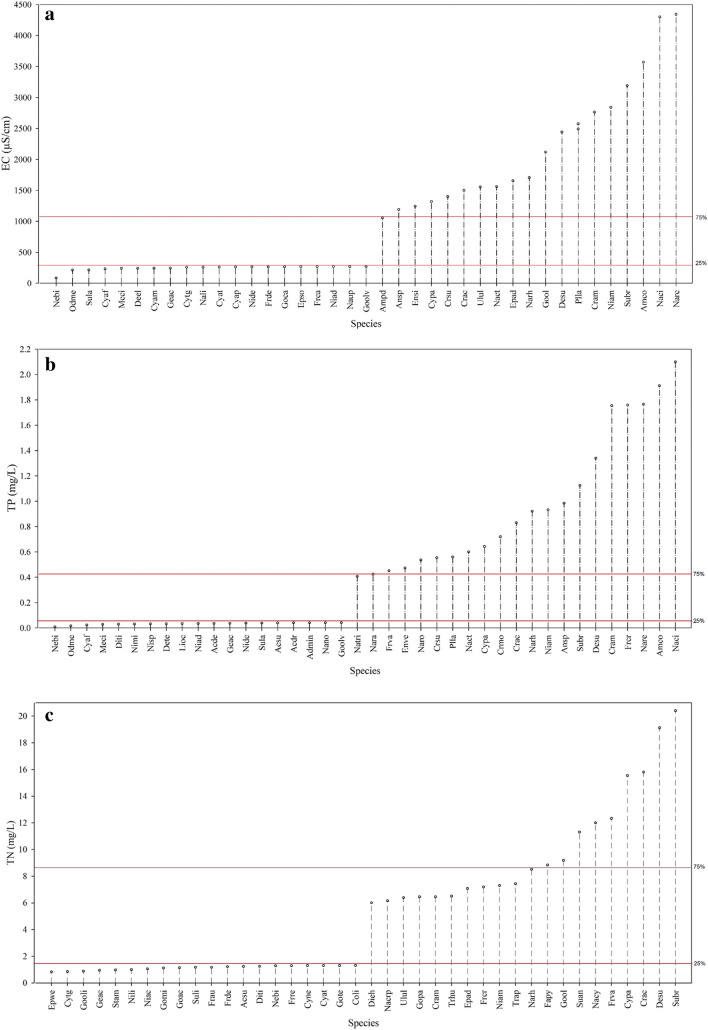

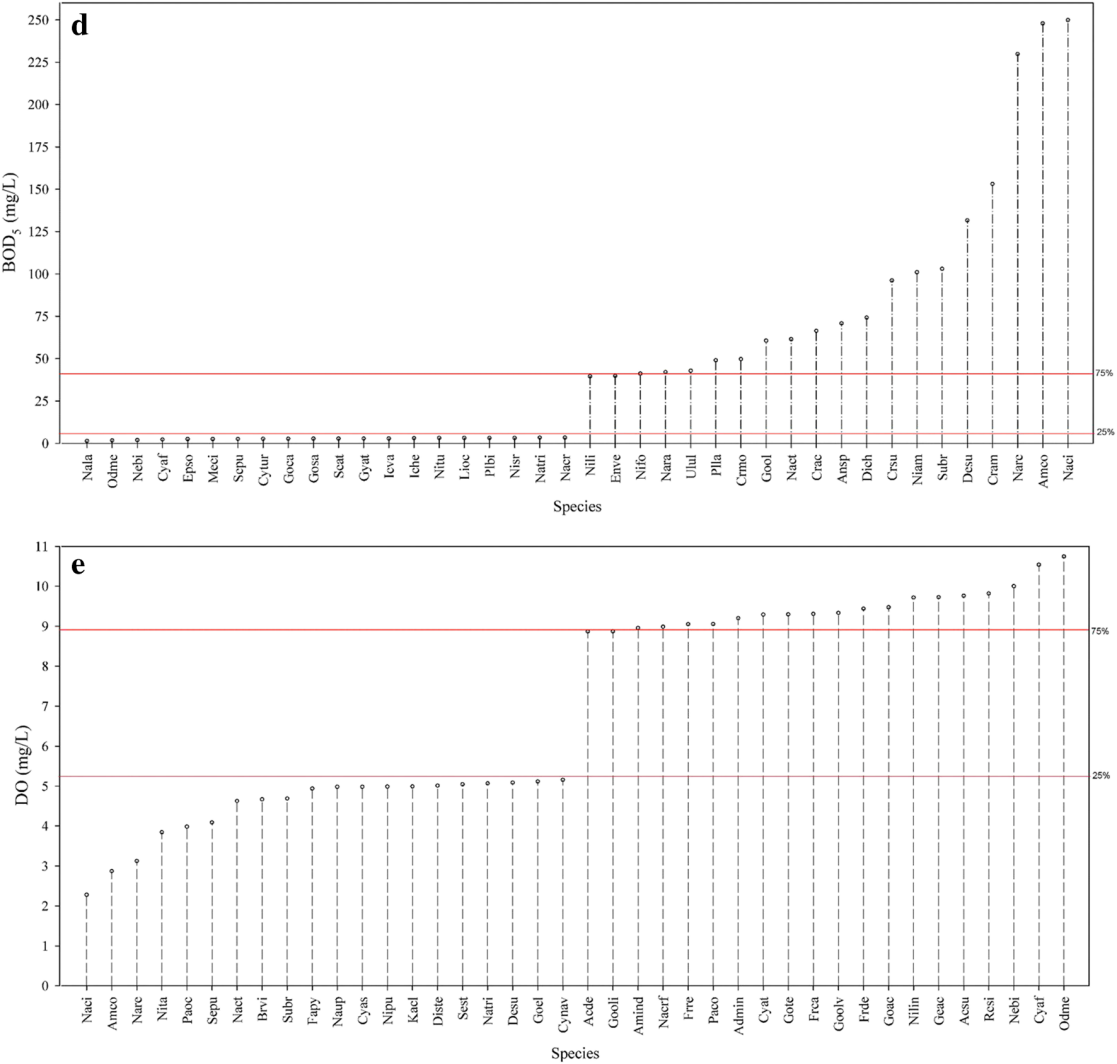
Optimum levels of diatom species for (**a**) electrical conductivity (EC), (**b**) total phosphorus (TP), (**c**) total nitrogen (TN), (**d**) biological oxygen demand (BOD_5_), and (**e**) dissolved oxygen (DO). Red lines on the plots indicate 25th and 75th percentiles. Species codes are represented in Supplementary 1

The results of the weighted average (WA) regression supported the findings of the CCA. Pollution-tolerant species characterized by Karasu (S06) and Erkenez (S07) streams had optimum levels larger than the 75th percentile for EC (1052 µS/cm, Fig. 4a), TP (0.42 mg/L, Fig. 4b), TN (8.55 mg/L, Fig. 4c), and BOD_5_ (40 mg/L) (Fig. 4d). Species, *Navicula recens*, *N. cincta*, *A. copulata*, *S. brebissoni*, *C. ambigua*, *N. amphibia*, *C. accomoda*, and *N. recens*, can tolerate pollution and their optimum levels were higher than the 75th percentile for EC (1152 µS/cm, Fig. 4a). On the other hand, pollution-sensitive species including *N. binodiformis*, *O. mesodon*, *M. circulare*, and *C. affinis* exhibited sub-optimal levels below the 25th percentile for EC (269 µS/cm, Fig. 4a). Pollution-sensitive species (e.g., *N. binodiformis*, *O. mesodon*, and *C. affinis*) showed optimum levels lower than the 25th percentile for TP (0.15 mg/L, Fig. 4b) and BOD_5_ gradients (3.1 mg/L, Fig. 4d). Pollution-tolerant species (e.g., *N. cincta*, *A. copulata*, *S. brebissoni*, *C. ambigua*, and *N. recens*) had optimum levels smaller than the 25th percentile for the DO gradient (0.98 mg/L) (Fig. 4e). On the other hand, pollution-sensitive species (e.g., *O. mesodon*, *C. affinis*, and *N. binodiformis*) had optimum levels higher than the 75th percentile for DO (9.40 mg/L, Fig. 4e).

### Bioassessment

**Fig. 5:**
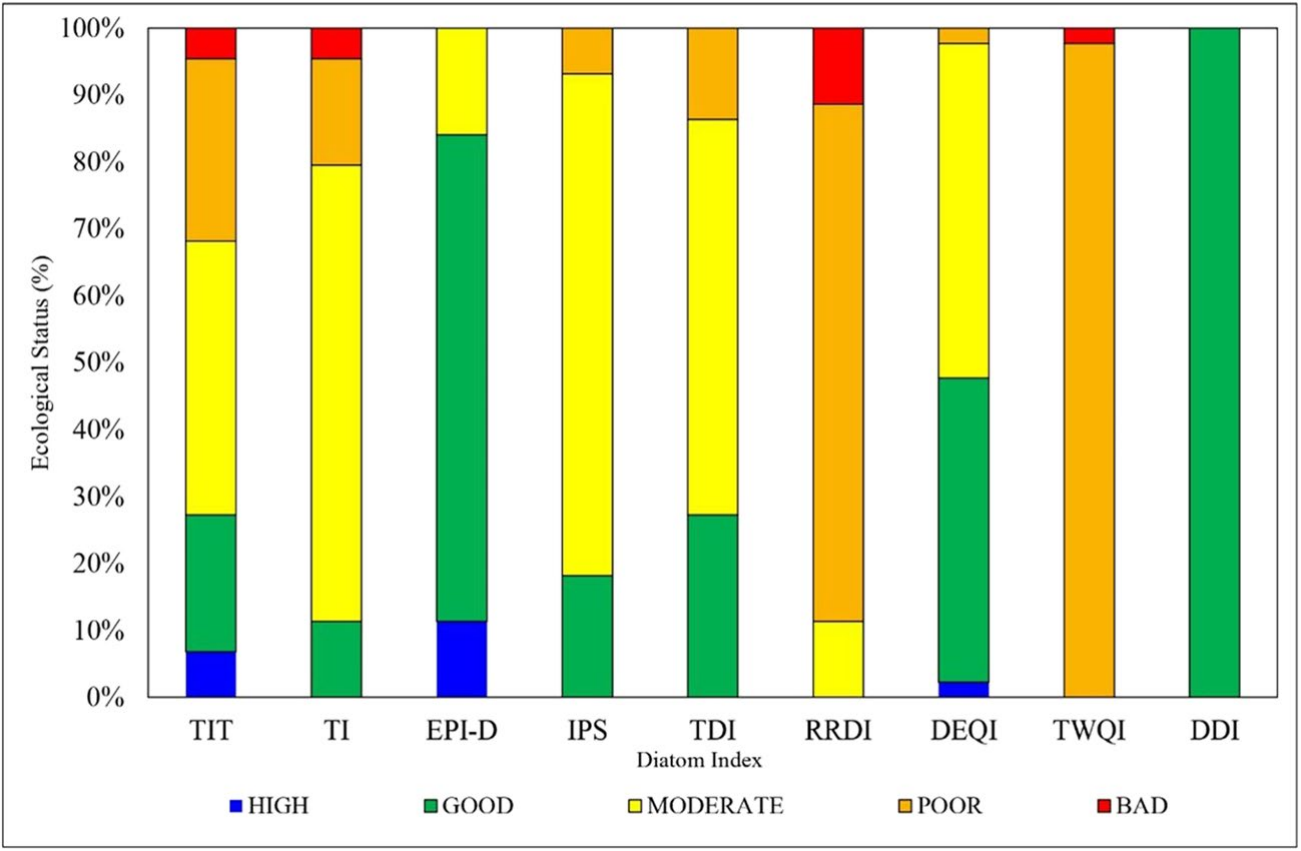
The percentages of ecological quality statuses specified by different diatom indices for the sampled streams in the Ceyhan River Basin. Abbreviations of diatom indices are given in Table 3

The bioassessment of sampling stations based on the results of various diatom indices is given in Table 3. Diatom indices developed in different ecoregions demonstrated distinct scores and ecological statuses ranging from high to poor for the studied streams in the Ceyhan River Basin. Figure 5 shows that among diatom indices, EPI-D showed that 11% of streams had a high ecological status. This was followed by TIT with 8% and DEQI with 3%. Others could not show high ecological statuses. TWQI and DDI could not differentiate the ecological status of the stations, but TWQI only presented a bad ecological status for S35. Similarly, RRDI made a small distinction in the environmental conditions of the sampling stations (for more, see Table 3).

**Table 3:**
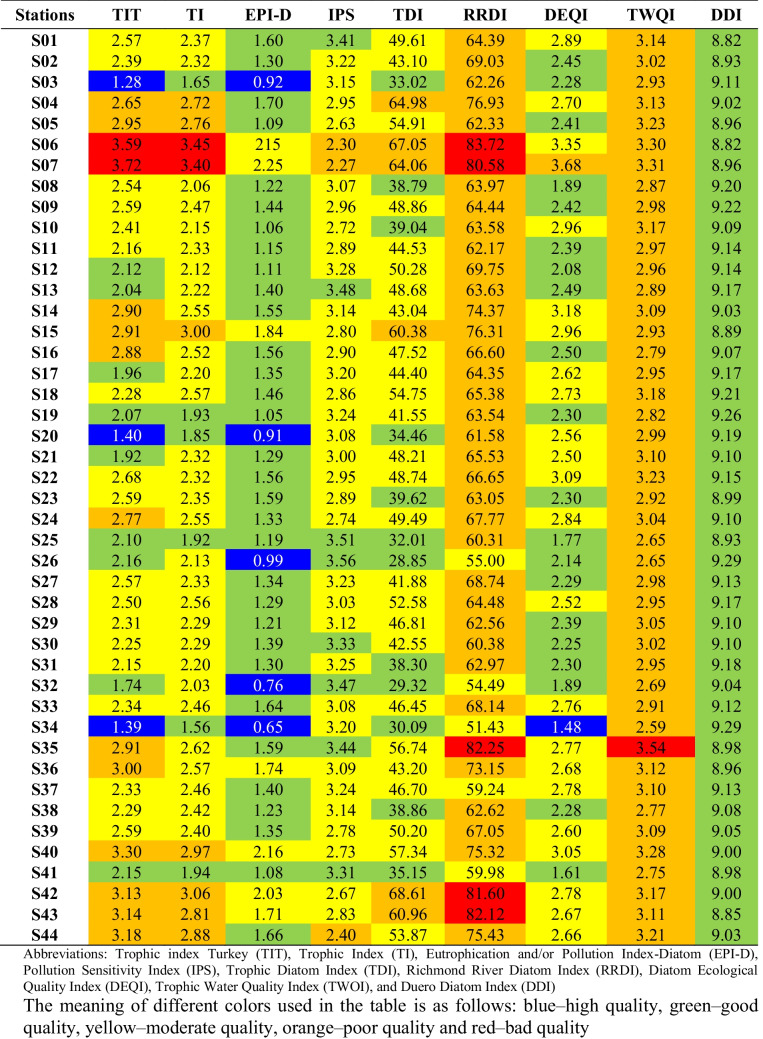
Different ecoregional diatom indices scores for the streams sampled in the Ceyhan River Basin

The TIT and EPI-D indices specified high ecological status for Aksu (S03), Yeşilgöz (S20), and Kandırın Streams (S34) (Table 3). The TIT, TI, and RRDI indices elucidated that the bad ecological situations of S06 and S07 related to extreme degradation. European indices showed similar behaviors for the bioassessment of streams in the Ceyhan River Basin. However, the TWQI (Brazil) and the DDI (Spain) indices could not distinguish the environmental conditions of the sampled streams.

Correlations between diatom-based indices and environmental variables are summarized in Table 4. Among the European diatom indices, TWQI and DEQI showed positively significant relationships with nutrients, while the IPS and DDI indices exhibited negatively significant associations. TP gradient demonstrated positive correlations with TIT (*r* = 0.820, *p* < 0.01), TI (*r* = 0.737, *p* < 0.01), EPI-D (*r* = 0.775, *p* < 0.01), TDI (*r* = 0.775, *p* < 0.01), TWQI (*r* = 0.565, *p* < 0.01), and DEQI (*r* = 0.549, *p* < 0.01), but a negative correlation with IPS (− *r* = 0.596, *p* < 0.01) and DDI (− *r* = 0.609, *p* < 0.01).

**Table 4:** Spearman correlation test results for the relationships between diatom-based indices and environmental variables

	TIT	TI	EPI-D	IPS	TDI	RRDI	DEQI	TWQI	DDI
TP	0.820**	0.737**	0.775**	− 0.596**	0.656**	0.749**	0.549**	0.565**	− 0.609**
TN	0.614**	0.577**	0.570**	− 0.570**	0.645**	0.655**	0.571**	0.691**	− 0.588**
DO	− 0.607**	− 0.611**	− 0.622**	0.408**	− 0.536**	− 0.433**	− 0.500**	− 0.523**	0.511**
BOD_5_	0.452**	0.275	0.217	− 0.384*	0.204	0.182	0.239	0.203	− 0.455**
EC	0.645**	0.510**	0.479**	− 0.369*	0.415**	0.371*	0.376*	0.440**	− 0.598**
SO_4_	0.566**	0.407**	0.463**	− 0.335*	0.292	0.362*	0.352*	0.289	− 0.405**
NO_3_	0.562**	0.485**	0.503**	− 0.461**	0.532**	0.589**	0.427**	0.601**	− 0.481**
NO_2_	0.621**	0.587**	0.755**	− 0.383*	0.588**	0.714**	0.542**	0.477**	− 0.440**
TOC	0.603**	0.482**	0.379*	− 0.466**	0.438**	0.457**	0.342*	0.289	− 0.225
Hardness	0.552**	0.435**	0.434**	− 0.318*	0.372*	0.292	0.320*	0.312*	− 0.512**
Temp	0.661**	0.589**	0.511**	− 0.355*	0.459**	0.433**	0.514**	0.571**	− 0.528**
Alt	− 0.591**	− 0.380*	− 0.412**	00.297	− 0.337*	− 0.398**	− 0.239	− 0.389**	0.450**
Al	0.465**	0.502**	0.589**	− 0.359*	0.380*	0.487**	0.403**	0.351*	− 0.335*
Fe	0.545**	0.578**	0.673**	− 0.367*	0.417**	0.580**	0.366*	0.311*	− 0.384*
B	0.499**	0.342*	0.265	− 0.316*	0.269	0.178	0.197	0.348*	− 0.428**
Cl	0.748**	0.592**	0.570**	− 0.396**	0.421**	0.461**	0.413**	0.458**	− 0.599**
K	0.738**	0.606**	0.566**	− 0.491**	0.516**	0.517**	0.495**	0.474**	− 0.517**
Na	0.671**	0.496**	0.521**	− 0.342*	0.331*	0.343*	0.298*	0.426**	− 0.570**

*TP* total phosphorus, *TN* total nitrogen, *DO* dissolved oxygen, *BOD*_*5*_ biological oxygen demand, *EC* electrical conductivity, *SO*_*4*_ sulfate, *NO*_*3*_ nitrate, *NO*_*2*_ nitrite, *TOC* total organic carbon, *Temp* temperature, *Alt* altitude, *Al* aluminum, *Fe* iron, *B* boron, *Cl* chlorine, *K* potassium, and *Na* sodium

* and ** represent significance levels at 0.05 and 0.001, respectively. Abbreviations of diatom indices are given in Table 3

The percentages of diatom species and their numbers of individuals (valves) used to compute scores for diatom indices are given in Supplementaries 4 and 5, respectively. The coverage percentages of species of diatom indices utilized in Ceyhan River streams varied depending on the sampled sites and the types of indices applied. European diatom indices showed higher percentages of diatom species compared to other ecoregional diatom indices. Among the indices, IPS exhibited the highest percentage of diatom species and numbers of individuals (valves).

## Discussion

Various streams in the Ceyhan River Basin exhibit distinct hydromorphological characteristics in the Mediterranean region, and anthropogenic activities have significant impacts on them. For example, the Karasu (S06) and Erkenez (S07) streams exhibited the most deterioration. They had high EC, BOD_5_, and TP values while having low DO values, which indicate class IV water quality (TSWQR 2016). Furthermore, the downstream sections (S39, S40, S43, and 44) of the Ceyhan River had relatively high EC values, corresponding class III water quality (TSWQR 2016). Overall ion concentration in the water is linked to the EC of aquatic ecosystems. It is also a valuable indicator for assessing the changes in water geochemistry caused by human activities, which makes it a widely accepted and effective monitoring parameter (Çelekli et al. 2019; Carol et al. 2023). Karasu (S06) and Erkenez (S07) streams, located within the industrial zone in Kahramanmaraş province, heavily experienced the discharge of especially textile factory waste. Besides, deterioration of water quality in downstream areas of the Ceyhan River except Karaçay stream (S41) can occur due to various factors and human activities. For example, increased erosion and sedimentation in upstream areas transport higher sediment loads to downstream. Excess nutrients from agricultural runoff, sewage discharges, and fertilizers are other pollution-related factors that enter the Ceyhan River downstream. Climate change can also affect downstream water quality through altered precipitation patterns, increased frequency of extreme weather events, and rising temperatures.

Different environmental conditions in streams strongly influenced the diatom composition in the Ceyhan River Basin. Understanding the environmental conditions that drive diatom composition in streams is crucial for assessing stream health, water quality, and ecological integrity. The CCA with the forward selection of Monte Carlo permutation test indicated that BOD_5_, TP, EC, DO, and TN played significant roles on the distribution of diatom species composition in the present study. Multivariate statistical analyses indicated that the deterioration of Karasu (S06) and Erkenez (S07) streams was closely related to high TP and EC levels and low DO values in the CCA diagram (Fig. 3a). These streams were characterized by pollution-tolerant species (Supplementary 1) like *N. cincta* (137), *N. recens* (152), *C. ambigua* (33), and *F. crotonensis* (91) (Fig. 3b). The results of WA supported this, indicating that the mentioned species had optima higher than the 75th percentile for EC, BOD_5_, and TP in the Ceyhan River Basin. Karasu (S06) and Erkenez (S07) streams had water with high turbidity under the pressure of industrial wastes, especially textile wastes. *Navicula recens* in Karasu (S06) and Erkenez (S07) streams was also commonly found in eutrophic rivers characterized by relatively high EC and TP levels (Çelekli et al. 2018; Chen et al. 2019; Hwang et al. 2023). Fazlutdinova et al. (2020) reported that *N. cincta* is a halophilic species that prefers mostly eutrophic, electrolyte-rich waters and was found in hot spring systems in Kamchatka. *Navicula cincta* was also found in carbonate spring habitats (Italy), where there is nutrient enrichment with high EC levels (Cantonati et al. 2016). *Craticula ambigua* shows distribution mostly in eutrophic conditions, and Alakananda et al. (2011) underlined the tolerance capacity of species to high pollution levels with high EC levels. The occurrence of *C. ambigua* in Karasu (S06) and Erkenez (S07) streams with high EC (Fig. 4a), TP (Fig. 4b), and BOD_5_ (Fig. 4d) levels supports the previous statements about the species.

Streams like Aksu Spring Brook, Gözpnar Creek, Göksun Creek, and Yeşilgöz Spring Brook (Fig. 3a) had a high oxygen content, low EC, and nutrient values. These streams were characterized by pollution-sensitive species (Supplementary 1) such as *Achnanthidium rivulare*, *Odontidium mesodon*, and *Cymbella affinis* (Fig. 3b). The ecological preference of *A. rivulare* for aquatic bodies with low calcium and nutrient salts was also recorded in the Boluo River, Erhai Basin of Southwestern China (Yang et al. 2013). It is known that *Achnanthidium* species are mainly primary colonizers in lotic ecosystems at high elevations with minimal channel stability (Fell et al. 2018). A few species of *Achnanthidium*, such as *A. minutissimum*, preferred least disturbed streams of mountainous areas in Spain (González-Paz et al. 2022) and Antalya River Basin (Çelekli et al. 2022a). *Odontidium mesodon* is another pollution-sensitive species in the present study, which has been documented in high-elevation streams influenced by rock glaciers in Switzerland (Peszek et al. 2022). It has distributions in clean, well-oxygenated, and low-nutrient conditions in different ecoregions (Rott et al. 1999; Dell’Uomo et al. 2004; Çelekli et al. 2019). Among pollution-sensitive species in the present study, *Cymbella affinis* was previously found in running water bodies with low nutrient concentrations (Çelekli and Bilgi 2019; Sushmitha and Mahesh 2021; Peszek et al. 2022), and it is also known as a pollution-sensitive species (Potapova et al. 2004; Delgado et al. 2012) with low trophic weight (Wang et al. 2014; Çelekli et al. 2019). The co-occurrence of *Achnanthidium*, *Odontidium*, and *Cymbella* in the least distributed streams in the Ceyhan River Basin was also emphasized in Alpine streams (Peszek et al. 2022) and springs (Cantonati et al. 2006) with low conductivity and nutrients in Switzerland. Cantonati et al. (2006) reported that *Achnanthes minutissima* (a synonym of *Achnanthidium minutissimum*) and *Diatoma mesodon* (a synonym of *Odontidium mesodon*) were the two most common and abundant species in the spring of the Alps, and *Achnanthes* was generally prefer silicate, whereas *Cymbella* was especially favored on carbonate. The presence of pollution-sensitive species indicates the health of the ecosystems and their presence is an indication that the water body they are in is not significantly impacted by pollution or other disturbances. Aksu Spring Brook, Gözpınar Creek, Göksun Creek, and Yeşilgöz Spring Brook are upstream areas in the Ceyhan River Basin where pollution-sensitive species were observed. Compared to downstream areas, upstream areas were experienced less impact from human activities, resulting in better water quality. This is because there may be fewer industrial and agricultural operations, reduced urbanization, and limited human settlements when compared to the downstream areas. As a result, less pollution and contamination enter the water bodies.

Diatom-based water quality studies employing a variety of diatom indices have become a standard approach to assessing lotic ecosystems (Rott et al. 1999; Lobo et al. 2015; Çelekli et al. 2022a). Of the indices used in the present study, the EPI-D had the highest percentage of high ecological status. Similar behavior was also found in the Antalya River Basin (Çelekli et al. 2022a) for the EPI-D. The Ceyhan and Antalya basins are in the Mediterranean region, where EPI-D was developed. Although DDI (Álvarez-Blanco et al. 2013) developed in Spain as a Mediterranean region, it could not distinguish the ecological status of the sampled streams and showed good ecological status for all stations in this study (Table 3). This situation was also observed in the Antalya (Çelekli et al. 2022a) and Konya Closed River basins (Çelekli et al. 2022b). According to Çelekli et al. (2021), DDI is not sensitive to the variations between sites since it hardly changes even though the environmental conditions vary. They reported that the class boundaries of the DDI strongly affect the bioassessment results. TWQI resulted in poor ecological status for all sampled streams except S35, which had a bad condition. This is because of the different ecoregional factors like climate, geology, land use, and anthropogenic activities in Brazil significantly affect the ecological preferences of diatom species, which led to the development of eco-region based index TWQI.

The current study revealed that TIT, TI, EPI-D, TDI, DEQI, and TWQI had significantly positive correlations with the TP gradient, while IPS and DDI had negative correlations. In the bio-assessment of sampling stations in the Ceyhan River Basin, European diatom indices mostly showed similar behavior. European diatom indices like TIT, TI, and EPI-D did not only show significant correlations with nutrients but also had a significantly positive correlation with EC, TOC, and metals. TP is the most effective environmental factor on the diatom species composition and is an important chemical variable in the calculation of the trophic weights of various diatom indices (Rott et al. 1999; Dell’Uomo et al. 2004; Çelekli et al. 2019). The correlation results with the TP gradient (see Table 4) indicated that TIT is suitable for the bio-evaluation of streams in the Ceyhan River Basin. TIT indicated bad ecological status in Karasu (S06) and Erkenez (S07) streams when poor ecological status was observed in downstream stations (S40-S44 stations except S40), and these findings were supported by the scores of the TI. On the other hand, TIT and EPI-D resulted in high ecological status for S03, S20, and S34. Previous studies (e.g., Çeleklı and Bilgi 2019; Çelekli et al. 2021; Lazaridou et al. 2018) reported that the specific hydrological and climatic conditions of this Mediterranean region affect the trophic weight of diatom taxa, making the European indices ineffective. This is because each metric uses different number of diatom species (Çelekli et al. 2022a, b). Therefore, the number of diatom species of the applied diatom indices does not encompass all the diatom species observed in the streams of the Ceyhan River, as a limiting factor to assess the water quality. European diatom indices utilized different mean percentages (e.g., IPS (69.8%), TI (53.3%), EPI-D (50.2%), and TIT (35.3%) in Supplementary 4) of diatoms species found in streams of the Ceyhan River Basin. Although TIT had the lowest species number to assess ecological status of streams in the Ceyhan River Basin, it was found to be a competitive index. In light of this information, ecoregionally specific diatom indices like TIT is required to make bioassessment more accurate due to the ecological preferences of diatom taxa. This is because variations in the floristic spectrum covered by each method frequently result in the ineffectiveness of each diatom index when diagnosing the ecological status of environments located outside of the biogeographic area where they were established (Hering et al. 2010; Çelekli et al. 2019). Estimating the geographical extension of ecosystems at regional scales is a fundamental aspect of biogeography (Matthews et al. 2017) and has led to the creation of ecoregions, defined as relatively large units of land containing a distinct assemblage. However, ecoregions defined by fish species presence/absence may not necessarily provide a more accurate description of the European bioregions overall (Tachos et al. 2023). Occurrences of other taxa with differing dispersal abilities may exhibit better correspondence with the currently used ecoregions, e.g., diatoms. To ensure the adequate prioritization of resources for the management and protection of European rivers, it is necessary to conduct an up-to-date ecoregional delimitation in European freshwaters.

## Conclusion

The present study’s findings underline the importance of diatom taxa as bioindicators to estimate the water quality of lotic systems. Multivariate statistical analyses pointed out that the distribution and composition of diatom species are regulated by the physicochemical variables in streams. Water temperature, TP, DO, BOD_5_, and TN displayed the greatest impacts on the distribution of diatom species composition. Although diatom indices showed a significant correlation with the TP gradient, they have different scores, resulting in ecological statuses ranging from bad to high in the Ceyhan River basin. Among the diatom indices, TIT was the most suitable metric to assess the ecological status of streams in the present study. TIT indicated deterioration of water quality in Karasu (S06), Erkenez (S07), and downstream areas of the Ceyhan River except Karaçay stream (S41), which are complementary to the environmental evaluation under the impact of human activities. The least deteriorated sampling stations in the basin were also distinguished by the TIT. Results indicated that the presence of different tolerant/sensitive diatom species in the tested diatom indices could influence the results of the bioassessment of the water quality of streams in the Ceyhan River Basin. Therefore, eco-region-based diatom indices, such as TIT, are required to assess the ecological status of streams more accurately in the Mediterranean region.

### Supplementary Information

Below is the link to the electronic supplementary material.

Supplementary file1 (DOCX 71 KB)

Supplementary file2 (DOCX 36 KB)

Supplementary file3 (DOCX 67 KB)

Supplementary file4 (DOCX 73 KB)

Supplementary file5 (DOCX 48 KB)

## Data Availability

Data will be made available on reasonable request.
