# Supplementary material for: Determination of ecological statuses of streams in the Ceyhan River Basin using composition and ecological characteristics of diatoms
Source: Environ Sci Pollut Res Int. 2024 May 7;31(23):34738–55. doi: 10.1007/s11356-024-33518-0 (PMC11136811; doi:10.1007/s11356-024-33518-0)
Supplement: Supplementary file 1 — Supplementary file1 (DOCX 71 KB) [file 11356_2024_33518_MOESM1_ESM.docx]

**Supplementary 1.** Diatom taxa list collected from the 44 streams in Ceyhan River Basin during three sampling periods (spring, summer and autumn). RL means red list. In the pollution column, S and T represent pollution-sensitive species and pollution-tolerant species, respectively (Spaulding et al., 2021).

| No | Code | Species | RL | Pollution |
| --- | --- | --- | --- | --- |
| 1 | Acat | *Achnanthidium atomoides* Monnier, Lange-Bertalot & Ector | * |  |
| 2 | Acca | *Achnanthidium catenatum* (Bily & Marvan) Lange-Bertalot | * |  |
| 3 | Acde | *Achnanthidium delmontii* Pérès, Le Cohu & Barthès | * |  |
| 4 | Acdr | *Achnanthidium druartii* Rimet & Couté | D |  |
| 5 | Aceu | *Achnanthidium eutrophilum* (Lange-Bertalot) Lange-Bertalot | * |  |
| 6 | Acex | *Achnanthidium exiguum* (Grunow) Czarnecki |  |  |
| 7 | Acgr | *Achnanthidium gracillimum* (F.Meister) Lange-Bertalot | 2 |  |
| 8 | Acmi | *Achnanthidium minutissimum* (Kützing) Czarnecki | D | S |
| 9 | Acpy | *Achnanthidium pyrenaicum* (Hustedt) H.Kobayasi | * |  |
| 10 | Acri | *Achnanthidium rivulare* Potapova & Ponader | * | S |
| 11 | Acsu | *Achnanthidium subatomoides* (Hustedt) O.Monnier, Lange-Bertalot & Ector |  |  |
| 12 | Acsub | *Achnanthidium sublineare* Van de Vijver, Jarlman & Ector |  |  |
| 13 | Admi | *Adlafia minuscula* (Grunow) Lange-Bertalot | * |  |
| 14 | Admin | *Adlafia minuscula* var. muralis (Grunow) Lange-Bertalot | * |  |
| 15 | Amco | *Amphora copulata* (Kützing) Schoeman & R.E.M.Archibald | * | T |
| 16 | Amin | *Amphora inariensis* Krammer | * | S |
| 17 | Amind | *Amphora indistincta* Levkov | * |  |
| 18 | Amov | *Amphora ovalis* (Kützing) Kützing | * | T |
| 19 | Ampd | *Amphora pediculus* (Kützing) Grunow | * | T |
| 20 | Ampe | *Amphipleura pellucida* (Kützing) Kützing | * |  |
| 21 | Amsp | *Amphora* sp*.* Ehrenberg ex Kützing |  |  |
| 22 | Amve | *Amphora vetula* Levkov |  |  |
| 23 | Ansp | *Anomoeoneis sphaerophora* Pfitzer | * |  |
| 24 | Auam | *Aulacoseira ambigua* (Grunow) Simonsen | * |  |
| 25 | Brsp | *Brachysira* sp*. Kützing* |  |  |
| 26 | Brvi | *Brachysira vitrea* (Grunow) R.Ross | 2 |  |
| 27 | Coeu | *Cocconeis placentula* var. euglypta (Ehrenberg) Cleve | * |  |
| 28 | Coeug | *Cocconeis euglyptoides* (Geitler) Lange-Bertalot | D |  |
| 29 | Coli | *Cocconeis lineata* Ehrenberg | * |  |
| 30 | Cope | *Cocconeis pediculus* Ehrenberg | * |  |
| 31 | Copl | *Cocconeis placentula* Ehrenberg | D |  |

| **Supplementary 1. Continued** | | |  |  |
| --- | --- | --- | --- | --- |
| No | Code | Species | RL | Pollution |
| 32 | Crac | *Craticula accomoda* (Hustedt) D.G.Mann | * | T |
| 33 | Cram | *Craticula ambigua* (Ehrenberg) D.G.Mann | * | T |
| 34 | Crmo | *Craticula molestiformis* (Hustedt) Mayama | * |  |
| 35 | Crsu | *Craticula subminuscula* (Manguin) C.E.Wetzel & Ector |  | T |
| 36 | Cyaf | *Cymbella affinis* Kützing | 2 | S |
| 37 | Cyam | *Cymbopleura amphicephala* (Nägeli ex Kützing) Krammer | G | S |
| 38 | Cyan | *Cymbella anglica* Lagerstedt |  |  |
| 39 | Cyap | *Cymatopleura apiculata* W.Smith |  |  |
| 40 | Cyas | *Cymbella aspera* (Ehrenberg) Cleve | G |  |
| 41 | Cyat | *Cyclotella atomus* Hustedt | * |  |
| 42 | Cyco | *Cymbella compacta* Østrup | * |  |
| 43 | Cyex | *Cymbella excisa* Kützing | * | S |
| 44 | Cyexc | *Cymbella excisiformis* Krammer | 3 |  |
| 45 | Cyhu | *Cymbella hustedtii* Krasske | G |  |
| 46 | Cyin | *Cyclostephanos invisitatus* E.C.Theriot, Stoermer & Håkanasson | * |  |
| 47 | Cyir | *Cyclotella iris* Brun & Héribaud |  |  |
| 48 | Cyku | *Cymbopleura kuelbsii* Krammer |  |  |
| 49 | Cyla | *Cymbella lanceolata* C.Agardh | 2 |  |
| 50 | Cyli | *Cyclotella litoralis* Lange & Syvertsen |  |  |
| 51 | Cyme | *Cyclotella meduanae* H.Germain | * |  |
| 52 | Cymg | *Stephanocyclus meneghinianus* (Kützing) Kulikovskiy, Genkal & Kociolek |  |  |
| 53 | Cyna | *Cymbopleura naviculiformis* (Auerswald ex Heiberg) Krammer | * |  |
| 54 | Cynav | *Cymbella naviculiformis* var. linearis Foged |  |  |
| 55 | Cyne | *Cymbella neocistula* Krammer | * |  |
| 56 | Cynl | *Cymbella neolanceolata* W.Silva | * |  |
| 57 | Cyoc | *Pantocsekiella ocellata* (Pantocsek) K.T.Kiss & Ács | * |  |
| 58 | Cypa | *Cymbella parva* (W.Smith) Kirchner | G | S |
| 59 | Cysu | *Cymbella subhelvetica* Krammer | 3 |  |
| 60 | Cytg | *Cymbella turgidula* Grunow |  | S |
| 61 | Cytu | *Cymbella tumidula* Grunow | * |  |

| **Supplementary 1. Continued** | | |  |  |
| --- | --- | --- | --- | --- |
| No | Code | Species | RL | Pollution |
| 62 | Cytur | *Cymbella turgida* W.Gregory |  |  |
| 63 | Deel | *Denticula elegans* Kützing | G |  |
| 64 | Desu | *Denticula subtilis* Grunow | * |  |
| 65 | Dete | *Denticula tenuis* Kützing | * | S |
| 66 | Dieh | *Diatoma ehrenbergii* Kützing | * | S |
| 67 | Dige | *Didymosphenia geminata* (Lyngbye) Mart.Schmidt | * | S |
| 68 | Dimo | *Diatoma moniliformis* (Kützing) D.M.Williams | * |  |
| 69 | Diob | *Diploneis oblongella* (Nägeli ex Kützing) A.Cleve | D |  |
| 70 | Diste | *Discostella stelligera* (Cleve & Grunow) Houk & Klee | * |  |
| 71 | Dite | *Diatoma tenuis* C.Agardh | * | S |
| 72 | Diti | *Diploneis tirolensis* Lange-Bertalot & Fuhrmann | D |  |
| 73 | Divu | *Diatoma vulgaris* Bory | * |  |
| 74 | Ence | *Encyonema cespitosum* Kützing |  |  |
| 75 | Enel | *Encyonema elginense* (Krammer) D.G.Mann | 2 |  |
| 76 | Enmc | *Encyonopsis microcephala* (Grunow) Krammer | * |  |
| 77 | Enmi | *Encyonopsis minuta* Krammer & E.Reichardt | D |  |
| 78 | Enmin | *Encyonema minutum* (Hilse) D.G.Mann | * | S |
| 79 | Ensi | *Encyonema silesiacum* (Bleisch) D.G.Mann | * |  |
| 80 | Ensu | *Encyonopsis subminuta* Krammer & E.Reichardt | G |  |
| 81 | Enve | *Encyonema ventricosum* (C.Agardh) Grunow | * |  |
| 82 | Epad | *Epithemia adnata* (Kützing) Brébisson | * |  |
| 83 | Epar | *Epithemia argus* (Ehrenberg) Kützing | * |  |
| 84 | Epgi | *Epithemia gibba* (Ehrenberg) Kützing |  | S |
| 85 | Epso | *Epithemia sorex* Kützing |  |  |
| 86 | Epwe | *Epithemia turgida* var. westermannii (Ehrenberg) Grunow |  |  |
| 87 | Fapy | *Fallacia pygmaea* (Kützing) Stickle & D.G.Mann | * |  |
| 88 | Fasu | *Fallacia subhamulata* (Grunow) D.G.Mann | * | T |
| 89 | Frau | *Fragilaria austriaca* (Grunow) Lange-Bertalot | G |  |
| 90 | Frca | *Fragilaria capucina* Desmazières |  |  |
| 91 | Frcr | *Fragilaria crotonensis* Kitton | * |  |

| **Supplementary 1. Continued** | | |  |  |
| --- | --- | --- | --- | --- |
| No | Code | Species | RL | Pollution |
| 92 | Frde | *Fragilaria delicatissima* Proshkina-Lavrenko |  |  |
| 93 | Frgr | *Fragilaria gracilis* Østrup | * |  |
| 94 | Frne | *Fragilaria nevadensis* J.E.Linares-Cuesta & P.M.Sánchez-Castillo |  |  |
| 95 | Frre | *Fragilaria recapitellata* Lange-Bertalot & Metzeltin | G |  |
| 96 | Frva | *Fragilaria vaucheriae* (Kützing) J.B.Petersen | * |  |
| 97 | Geac | *Geissleria acceptata* (Hustedt) Lange-Bertalot & Metzeltin | * |  |
| 98 | Gede | *Navigeia decussis* (Østrup) Bukhtiyarova |  | T |
| 99 | Goac | *Gomphonema acuminatum* Ehrenberg | G |  |
| 100 | Goca | *Gomphonella calcarea* (Cleve) R.Jahn & N.Abarca |  |  |
| 101 | Gocr | *Gomphonema cristatum* Ralfs |  |  |
| 102 | Gocu | *Gomphonema cuneolus* E.Reichardt | G |  |
| 103 | Gocur | *Gomphonema curvipedatum* H.Kobayasi ex K.Osada |  |  |
| 104 | Goel | *Gomphonema elegantissimum* E.Reichardt & Lange-Bertalot | * |  |
| 105 | Goin | *Gomphonema incognitum* E.Reichardt, Jüttner & E.J.Cox |  |  |
| 106 | Goit | *Gomphonema italicum* Kützing | * |  |
| 107 | Gola | *Gomphonema lagenula* Kützing |  |  |
| 108 | Golat | *Gomphonema lateripunctatum* E.Reichardt & Lange-Bertalot | V |  |
| 109 | Gomi | *Gomphonema micropus* Kützing | * |  |
| 110 | Gomin | *Gomphonema minutum* (C.Agardh) C.Agardh | * |  |
| 111 | Gomn | *Gomphonema minuta* P.Fusey |  |  |
| 112 | Gool | *Gomphonella olivacea* (Hornemann) Rabenhorst |  |  |
| 113 | Gooli | *Gomphonema olivaceoides* Hustedt | * |  |
| 114 | Gosp | *Gomphonella sp* Rabenhorst |  |  |
| 115 | Gopa | *Gomphonema parvulum* (Kützing) Kützing | * | T |
| 116 | Gopu | *Gomphonema pumilum* (Grunow) E.Reichardt & Lange-Bertalot | * |  |
| 117 | Gopy | *Gomphonema pygmaeum* J.Kociolek & E.Stoermer |  |  |
| 118 | Gorh | *Gomphoneis rhombica* (Fricke) Merino, García, Hernández-Mariné & Fernández |  |  |
| 119 | Gosa | *Gomphonema sarcophagus* W.Gregory | V |  |
| 120 | Gote | *Gomphonema tergestinum* (Grunow) Fricke | * |  |
| 121 | Gotu | *Gomphonema truncatum* var. turgidum (Ehrenberg) R.M.Patrick |  |  |

| **Supplementary 1. Continued** | | |  |  |
| --- | --- | --- | --- | --- |
| No | Code | Species | RL | Pollution |
| 122 | Grta | *Grunowia tabellaria* (Grunow) Rabenhorst |  |  |
| 123 | Gyac | *Gyrosigma acuminatum* (Kützing) Rabenhorst | * | T |
| 124 | Gyat | *Gyrosigma attenuatum* (Kützing) Rabenhorst | * |  |
| 125 | Haar | *Hannaea arcus* (Ehrenberg) R.M.Patrick | V | S |
| 126 | Hihu | *Hippodonta hungarica* (Grunow) Lange-Bertalot, Metzeltin & Witkowski | * |  |
| 127 | Iche | *Iconella helvetica* (Brun) Ruck & Nakov |  |  |
| 128 | Icva | *Iconella variabilis* Jurilj |  |  |
| 129 | Kacl | *Karayevia clevei* (Grunow) Bukhtiyarova | * |  |
| 130 | Kopa | *Kobayasiella parasubtilissima* (H.Kobayasi & T.Nagumo) Lange-Bertalot | V |  |
| 131 | Lehu | *Lemnicola hungarica* (Grunow) Round & Basson | * | T |
| 132 | Lioc | *Lindavia ocellata* (Pantocsek) K.T.Kiss & Ács | * |  |
| 133 | Meci | *Meridion circulare* (Greville) C.Agardh | * | S |
| 134 | Meva | *Melosira varians* C.Agardh | * | T |
| 135 | Naan | *Navicula antonii* Lange-Bertalot | * |  |
| 136 | Naca | *Navicula cari* Ehrenberg | * | T |
| 137 | Naci | *Navicula cincta* (Ehrenberg) Ralfs | * | T |
| 138 | Nacr | *Navicula cryptocephala* Kützing | * |  |
| 139 | Nacrf | *Navicula cryptofallax* Lange-Bertalot & G.Hofmann | * |  |
| 140 | Nacrp | *Navicula cryptocephala* var. veneta (Kützing) Rabenhorst |  |  |
| 141 | Nacrt | *Navicula cryptotenelloides* Lange-Bertalot | * |  |
| 142 | Nacry | *Navicula cryptotenella* Lange-Bertalot | * |  |
| 143 | Nact | *Navicula capitatoradiata* H.Germain ex Gasse | * |  |
| 144 | Nacy | *Navicula cryptocephaloides* Hustedt |  |  |
| 145 | Nade | *Navigeia decussis* (Østrup) Bukhtiyarova |  |  |
| 146 | Nagr | *Navicula gregaria* Donkin | * |  |
| 147 | Nala | *Navicula lanceolata* Ehrenberg | * |  |
| 148 | Nali | *Navicula libonensis* Schoeman | * |  |
| 149 | Nano | *Navicula novaesiberica* Lange-Bertalot, nom. inval. | * |  |
| 150 | Napa | *Navicula paraobesa* Metzeltin & Z.Levkov |  |  |
| 151 | Nara | *Navicula radiosa* Kützing | * |  |

| **Supplementary 1. Continued** | | |  |  |
| --- | --- | --- | --- | --- |
| No | Code | Species | RL | Pollution |
| 152 | Nare | *Navicula recens* (Lange-Bertalot) Lange-Bertalot | * | T |
| 153 | Narh | *Navicula rhynchocephala* Kützing | * |  |
| 154 | Naro | *Navicula rostellata* Kützing | * |  |
| 155 | Natr | *Navicula tripunctata* (O.F.Müller) Bory | * | T |
| 156 | Natri | *Navicula tridentula* Krasske | G |  |
| 157 | Natv | *Navicula trivialis* Lange-Bertalot | * | T |
| 158 | Naup | *Navicula upsaliensis* (Grunow) M.Peragallo | * |  |
| 159 | Navi | *Navicula vilaplanii* (Lange-Bertalot & Sabater) Lange-Bertalot & Sabater | * |  |
| 160 | Nebi | *Neidiomorpha binodiformis* M.Cantonati, Lange-Bertalot & N.Angeli | G | S |
| 161 | Niac | *Nitzschia acicularis* (Kützing) W.Smith | * |  |
| 162 | Niad | *Nitzschia tubicola* Grunow | * |  |
| 163 | Niam | *Nitzschia amphibia* Grunow | * | T |
| 164 | Nico | *Nitzschia costei* Tudesque, Rimet & Ector |  |  |
| 165 | Nide | *Nitzschia denticula* Grunow | V |  |
| 166 | Nidi | *Nitzschia dissipata* (Kützing) Rabenhorst | * |  |
| 167 | Nifo | *Nitzschia fonticola* (Grunow) Grunow | * |  |
| 168 | Niin | *Nitzschia inconspicua* Grunow |  |  |
| 169 | Nili | *Nitzschia liebethruthii* Rabenhorst |  |  |
| 170 | Nilin | *Nitzschia linearis* W.Smith | * |  |
| 171 | Nimi | *Nitzschia microcephala* Grunow | * |  |
| 172 | Nipa | *Nitzschia palea* (Kützing) W.Smith | * | T |
| 173 | Nipe | *Nitzschia perminuta* Grunow | * |  |
| 174 | Nipu | *Nitzschia puriformis* Hlúbiková & Ector | D |  |
| 175 | Nisi | *Nitzschia sigmoidea* (Nitzsch) W.Smith | * | T |
| 176 | Niso | *Nitzschia soratensis* E.A.Morales & M.L.Vis | * |  |
| 177 | Nisoc | *Nitzschia sociabilis* Hustedt | * |  |
| 178 | Nisp | *Nitzschia sp.* Hassall |  |  |
| 179 | Nita | *Grunowia tabellaria* (Grunow) Rabenhorst |  |  |
| 180 | Nitu | *Nitzschia tubicola* Grunow | * |  |
| 181 | Odme | *Odontidium mesodon* (Kützing) Kützing |  | S |

| **Supplementary 1. Continued** | | |  |  |
| --- | --- | --- | --- | --- |
| No | Code | Species | RL | Pollution |
| 182 | Paco | *Pantocsekiella comensis* (Grunow) K.T.Kiss & E.Ács |  |  |
| 183 | Pasp | *Pantocsekiella sp.* (Pantocsek) K.T.Kiss & Ács | * |  |
| 184 | Pivi | *Pinnularia viridis* (Nitzsch) Ehrenberg | D |  |
| 185 | Plbi | *Planothidium biporomum* (M.H.Hohn & Hellerman) Lange-Bertalot | D |  |
| 186 | Pldu | *Planothidium dubium* (Grunow) Round & Bukhtiyarova | * |  |
| 187 | Plfr | *Planothidium frequentissimum* (Lange-Bertalot) Lange-Bertalot | * |  |
| 188 | Plla | *Planothidium lanceolatum* (Brébisson ex Kützing) Lange-Bertalot | * |  |
| 189 | Psbr | *Pseudostaurosira brevistriata* (Grunow) D.M.Williams & Round | * |  |
| 190 | Resi | *Reimeria sinuata* (W.Gregory) Kociolek & Stoermer | * |  |
| 191 | Reun | *Reimeria uniseriata* Sala, Guerrero & Ferrario | * | T |
| 192 | Rhab | *Rhoicosphenia abbreviata* (C.Agardh) Lange-Bertalot | * |  |
| 193 | Rhgi | *Rhopalodia gibba* (Ehrenberg) O.Müller | * | T |
| 194 | Seat | *Sellaphora atomoides* (Grunow) Wetzel & Van de Vijver |  |  |
| 195 | Seca | *Sellaphora capitata* D.G.Mann & S.M.McDonald | D |  |
| 196 | Sepu | *Sellaphora pupula* (Kützing) Mereschkovsky | D |  |
| 197 | Sest | *Sellaphora stroemii* (Hustedt) H.Kobayasi | 2 |  |
| 198 | Stam | *Stauroneis amphicephala* Kützing | D |  |
| 199 | Stse | *Stauroneis separanda* Lange-Bertalot & Werum | V |  |
| 200 | Stsm | *Stauroneis smithii* Grunow | * |  |
| 201 | Stsp | *Stauroneis sp.* Ehrenberg, C.G. |  |  |
| 202 | Suan | *Surirella angusta* Kützing | * | T |
| 203 | Subr | *Surirella brebissonii* Krammer & Lange-Bertalot | * | T |
| 204 | Sula | *Surirella lacrimula* J.D.English | D |  |
| 205 | Suli | *Surirella librile* (Ehrenberg) Ehrenberg |  |  |
| 206 | Trap | *Tryblionella apiculata* W.Gregory |  |  |
| 207 | Trhu | *Tryblionella hungarica* (Grunow) Frenguelli |  | T |
| 208 | Trsp | *Tryblionella sp.* Smith, W. |  |  |
| 209 | Ulac | *Ulnaria acus* (Kützing) Aboal | * |  |
| 210 | Ulda | *Ulnaria danica* (Kützing) Compère & Bukhtiyarova | D |  |
| 211 | Ulul | *Ulnaria ulna* (Nitzsch) Compère | * | T |

RL: Red List, *: Number of species safe, **: Number of species is sufficient and safe, D: Not threatened, G: Hazard of unknown extent, V: Pre-warning list, 2: Highly endangered, 3: Endangered.

Spaulding SA, Potapova MG, Bishop IW, Lee SS, Gasperak TS, Jovanoska E, Edlund MB (2021) Diatoms. org: supporting taxonomists, connecting communities. Diatom Research, 36(4):291-304.https://doi.org/10.1080/0269249X.2021.2006790
