# Supplementary material for: Determination of ecological statuses of streams in the Ceyhan River Basin using composition and ecological characteristics of diatoms
Source: Environ Sci Pollut Res Int. 2024 May 7;31(23):34738–55. doi: 10.1007/s11356-024-33518-0 (PMC11136811; doi:10.1007/s11356-024-33518-0)
Supplement: Supplementary file 2 — Supplementary file2 (DOCX 36 KB) [file 11356_2024_33518_MOESM2_ESM.docx]

**Supplementary 2.** The class boundaries of different ecoregional diatom indices. For TIT, A1, A2, and A3 indicate three elevation levels; (0-800 m) (>800-1600 m) (>1600 m), respectively.

| Ecological Status | HIGH | GOOD | | MODERATE | | POOR | | BAD | Citation |
| --- | --- | --- | --- | --- | --- | --- | --- | --- | --- |
| Indices | **(<)** | **(≥)** | **(<)** | **(≥)** | **(<)** | **(≥)** | **(<)** | **(≥)** |  |
| **TIT A1** | 1.65 | 1.65 | 2.20 | 2.20 | 2.70 | 2.70 | 3.30 | 3.30 | (Çelekli et al. 2019) |
| **TIT A2** | 1.60 | 1.60 | 2.10 | 2.10 | 2.60 | 2.60 | 3.10 | 3.10 | (Çelekli et al. 2019) |
| **TIT A3** | 1.55 | 1.55 | 2.05 | 2.05 | 2.55 | 2.55 | 3.05 | 3.05 | (Çelekli et al. 2019) |
| **TI** | 1.4 | 1.4 | 2.0 | 2.0 | 2.7 | 2.7 | 3.3 | 3.3 | (Rott et al. 1999) |
| **EPI-D** | 1.0 | 1.0 | 1.7 | 1.7 | 2.3 | 2.3 | 3.0 | 3.0 | (Dell’Uomo 2004)) |
| **IPS** | 1.3 | 1.3 | 2.4 | 2.4 | 3.3 | 3.3 | 4.2 | 4.2 | (Coste 1982) |
| **TDI** | 20 | 20 | 40 | 40 | 60 | 60 | 80 | 80 | (Kelly et al. 2008) |
| **RRDI** | 20 | 20 | 40 | 40 | 60 | 60 | 80 | 80 | (Oeding and Taffs 2017) |
| **DEQI** | 1.5 | 1.5 | 2.5 | 2.5 | 3.5 | 3.5 | 4.5 | 4.5 | (Salinas-Camarillo et al. 2021) |
| **DDI** | (≥)10.0 | 9.99 | 8.50 | 8.49 | 7.50 | 7.49 | 6.00 | <6.00 | (Álvarez-Blanco et al. 2013) |
