# Supplementary material for: Determination of ecological statuses of streams in the Ceyhan River Basin using composition and ecological characteristics of diatoms
Source: Environ Sci Pollut Res Int. 2024 May 7;31(23):34738–55. doi: 10.1007/s11356-024-33518-0 (PMC11136811; doi:10.1007/s11356-024-33518-0)
Supplement: Supplementary file 3 — Supplementary file3 (DOCX 67 KB) [file 11356_2024_33518_MOESM3_ESM.docx]

**Supplementary 3.** Mean and standard deviations of physico-chemical variables measured from the sampled streams (St.) in the Ceyhan River Basin during three sampling periods (spring, summer and autumn).

| **St.** | **pH** | **SO_4_** | **NO_3_** | **NO_2_** | **NH_4_** | **TOC** | **Al** |
| --- | --- | --- | --- | --- | --- | --- | --- |
|  |  | mg/L | mg/L | mg/L | mg/L | mg/L | mg/L |
| **S01** | 7.81±0.36 | 19.98±0.78 | 18.17±10.85 | 0.03±0.03 | 0.05±0.00 | 0.54±0.12 | 0.01±0.01 |
| **S02** | 8.13±0.34 | 22.86±3.67 | 6.34±3.51 | 0.04±0.02 | 0.03±0.03 | 1.02±0.47 | 0.03±0.03 |
| **S03** | 8.05±0.15 | 6.48±0.44 | 3.80±2.08 | 0.03±0.03 | 0.04±0.02 | 0.35±0.16 | 0.02±0.00 |
| **S04** | 8.35±0.40 | 9.87±0.33 | 3.85±0.19 | 0.03±0.03 | 0.05±0.00 | 1.20±0.10 | 0.03±0.02 |
| **S05** | 8.60±0.00 | 15.60±0.00 | 4.23±0.00 | 0.00±0.00 | 0.05±0.00 | 1.50±0.00 | 0.01±0.00 |
| **S06** | 8.21±0.16 | 314.85±109.46 | 4.58±5.77 | 0.04±0.02 | 0.68±1.13 | 12.99±17.34 | 0.08±0.14 |
| **S07** | 7.82±0.35 | 141.38±12.06 | 2.21±2.96 | 0.07±0.03 | 0.53±0.85 | 10.01±6.09 | 0.28±0.30 |
| **S08** | 8.16±0.31 | 31.08±3.94 | 3.37±0.42 | 0.04±0.02 | 0.06±0.01 | 2.79±0.88 | 0.01±0.01 |
| **S09** | 8.44±0.21 | 27.01±14.55 | 3.46±1.53 | 0.03±0.03 | 0.03±0.03 | 1.80±0.35 | 0.02±0.01 |
| **S10** | 9.30±0.00 | 17.40±0.00 | 2.65±0.00 | 0.01±0.00 | 0.05±0.00 | 1.70±0.00 | 0.02±0.00 |
| **S11** | 8.53±0.20 | 18.58±1.85 | 0.77±0.52 | 0.03±0.02 | 0.04±0.01 | 1.22±0.24 | 0.04±0.02 |
| **S12** | 8.12±0.14 | 7.73±0.63 | 2.52±0.41 | 0.03±0.02 | 0.04±0.02 | 0.49±0.18 | 0.01±0.00 |
| **S13** | 8.59±0.36 | 7.20±3.31 | 1.07±0.74 | 0.05±0.00 | 0.09±0.08 | 1.25±0.38 | 0.02±0.01 |
| **S14** | 8.37±0.55 | 48.34±29.14 | 1.86±0.27 | 0.05±0.02 | 0.05±0.01 | 1.82±0.31 | 0.02±0.00 |
| **S15** | 8.84±0.05 | 57.64±30.92 | 1.76±1.78 | 0.07±0.02 | 0.21±0.22 | 1.83±0.74 | 0.05±0.04 |
| **S16** | 8.52±0.52 | 18.78±1.60 | 3.32±0.77 | 0.04±0.01 | 0.05±0.00 | 1.44±0.04 | 0.05±0.04 |
| **S17** | 8.31±0.20 | 20.03±9.78 | 2.09±0.17 | 0.03±0.02 | 0.04±0.02 | 0.53±0.14 | 0.02±0.01 |
| **S18** | 8.32±0.18 | 14.57±3.88 | 2.96±0.19 | 0.03±0.03 | 0.27±0.41 | 0.73±0.33 | 0.03±0.02 |
| **S19** | 8.18±0.10 | 12.50±1.13 | 2.47±1.81 | 0.03±0.03 | 0.04±0.02 | 12.55±21.18 | 0.01±0.00 |
| **S20** | 7.92±0.20 | 6.32±1.06 | 2.12±0.41 | 0.03±0.03 | 0.04±0.02 | 0.30±0.06 | 0.02±0.01 |
| **S21** | 8.13±0.19 | 10.00±1.84 | 3.34±0.60 | 0.07±0.06 | 0.11±0.10 | 0.76±0.06 | 0.02±0.01 |
| **S22** | 8.29±0.18 | 21.04±4.52 | 2.40±0.42 | 0.04±0.02 | 0.06±0.02 | 1.13±0.05 | 0.04±0.03 |
| **S23** | 8.34±0.09 | 18.94±11.51 | 4.36±2.47 | 0.03±0.03 | 0.05±0.00 | 0.66±0.08 | 0.13±0.13 |
| **S24** | 8.44±0.16 | 5.57±0.28 | 2.89±0.83 | 0.03±0.02 | 0.04±0.01 | 0.40±0.04 | 0.01±0.00 |
| **S25** | 8.24±0.08 | 18.39±0.85 | 0.49±0.23 | 0.03±0.04 | 0.05±0.00 | 0.92±0.02 | 0.02±0.03 |
| **S26** | 8.30±0.00 | 31.88±0.00 | 0.33±0.00 | 0.00±0.00 | 0.05±0.00 | 1.00±0.00 | 0.01±0.00 |
| **S27** | 8.18±0.12 | 21.64±12.17 | 1.19±0.32 | 0.03±0.03 | 0.04±0.01 | 0.89±0.09 | 0.02±0.01 |
| **S28** | 8.10±0.14 | 9.15±3.39 | 1.67±0.17 | 0.02±0.03 | 0.05±0.01 | 1.53±1.17 | 0.03±0.00 |
| **S29** | 8.23±0.25 | 7.90±1.82 | 0.90±0.79 | 0.03±0.02 | 0.04±0.01 | 0.95±0.22 | 0.02±0.01 |
| **S30** | 8.18±0.16 | 5.38±1.70 | 2.08±0.69 | 0.03±0.02 | 0.05±0.01 | 0.66±0.29 | 0.02±0.01 |
| **S31** | 8.13±0.03 | 8.48±1.64 | 0.79±0.39 | 0.03±0.03 | 0.05±0.01 | 0.66±0.19 | 0.03±0.01 |
| **S32** | 8.00±0.00 | 15.65±0.00 | 2.61±0.00 | 0.01±0.00 | 0.05±0.00 | 0.90±0.00 | 0.01±0.00 |
| **S33** | 8.29±0.09 | 6.64±0.69 | 2.09±0.29 | 0.03±0.02 | 0.05±0.02 | 0.71±0.04 | 0.02±0.02 |
| **S34** | 8.00±0.00 | 13.39±0.00 | 0.04±0.00 | 0.00±0.00 | 0.05±0.00 | 0.90±0.00 | 0.01±0.00 |
| **S35** | 8.16±0.14 | 11.27±1.63 | 5.14±2.57 | 0.04±0.01 | 0.05±0.01 | 1.16±0.49 | 0.03±0.04 |
| **S36** | 8.08±0.08 | 21.07±8.79 | 16.91±12.55 | 0.06±0.03 | 0.05±0.00 | 1.06±0.24 | 0.07±0.08 |
| **S37** | 8.33±0.10 | 57.87±47.83 | 1.48±0.12 | 0.03±0.02 | 0.05±0.01 | 0.77±0.46 | 0.02±0.01 |
| **S38** | 8.23±0.13 | 3.91±0.93 | 0.54±0.26 | 0.03±0.02 | 0.04±0.01 | 0.78±0.13 | 0.01±0.01 |
| **S39** | 8.14±0.05 | 50.48±4.68 | 14.04±8.14 | 0.05±0.00 | 0.05±0.00 | 1.05±0.20 | 0.01±0.01 |
| **S40** | 8.29±0.17 | 52.17±3.91 | 11.38±4.85 | 0.14±0.08 | 1.26±2.10 | 2.13±0.77 | 0.01±0.01 |
| **S41** | 8.50±0.20 | 51.56±17.00 | 0.44±0.10 | 0.03±0.03 | 0.05±0.00 | 0.72±0.09 | 0.01±0.01 |
| **S42** | 8.14±0.29 | 35.88±1.96 | 6.71±2.46 | 0.13±0.09 | 0.05±0.00 | 2.16±0.74 | 0.01±0.01 |
| **S43** | 7.99±0.02 | 82.42±39.12 | 120.99±103.86 | 0.43±0.39 | 0.05±0.00 | 1.75±0.55 | 0.13±0.12 |
| **S44** | 7.81±0.41 | 42.37±4.77 | 10.23±1.10 | 0.16±0.02 | 0.13±0.08 | 1.58±0.28 | 0.12±0.11 |

**Supplementary 2. Continued**

| **St.** | **F** | **Brm** | **Fe** | **Ca** | **Cl** | **Mg** | **K** | **Na** |
| --- | --- | --- | --- | --- | --- | --- | --- | --- |
|  | mg/L | mg/L | mg/L | mg/L | mg/L | mg/L | mg/L | mg/L |
| **S01** | 0.09±0.06 | 2.00±1.73 | 0.00±0.00 | 67.11±1.65 | 8.99±0.20 | 30.75±0.91 | 1.33±0.26 | 8.01±0.60 |
| **S02** | 0.08±0.07 | 2.00±1.73 | 0.02±0.02 | 55.15±6.77 | 4.90±0.79 | 18.97±1.36 | 1.35±0.37 | 7.03±0.55 |
| **S03** | 0.03±0.00 | 2.00±1.73 | 0.00±0.00 | 45.45±2.20 | 1.18±0.11 | 7.43±1.47 | 0.29±0.09 | 1.03±0.23 |
| **S04** | 0.11±0.06 | 2.00±1.73 | 0.02±0.02 | 67.34±11.99 | 4.91±1.29 | 18.34±1.57 | 1.04±0.18 | 5.78±0.20 |
| **S05** | 0.10±0.00 | nd | nd | 54.10±0.00 | 10.51±0.00 | 38.30±0.00 | 1.78±0.00 | 11.80±0.00 |
| **S06** | 0.04±0.06 | 2.00±1.73 | 0.07±0.10 | 82.20±28.69 | 253.26±237.69 | 58.39±30.19 | 28.26±11.14 | 368.6±468.44 |
| **S07** | 3.68±6.37 | 2.00±1.73 | 0.08±0.08 | 103.30±43.91 | 192.36±242.97 | 26.15±6.63 | 12.02±4.87 | 156.25±202.56 |
| **S08** | 0.08±0.01 | 2.00±1.73 | 0.00±0.01 | 53.64±2.08 | 5.63±1.10 | 13.99±0.34 | 1.16±0.11 | 6.58±0.89 |
| **S09** | 0.06±0.01 | 2.00±1.73 | 0.01±0.01 | 50.72±14.41 | 4.26±2.93 | 17.31±5.09 | 0.94±0.44 | 3.95±2.41 |
| **S10** | 0.05±0.00 | 0.00±0.00 | nd | 24.40±0.00 | 3.98±0.00 | 21.20±0.00 | 0.99±0.00 | 4.20±0.00 |
| **S11** | 0.04±0.03 | 2.00±1.73 | 0.01±0.00 | 32.33±2.21 | 3.19±0.82 | 28.12±13.79 | 0.88±0.17 | 5.59±2.04 |
| **S12** | 0.04±0.01 | 2.00±1.73 | nd | 42.11±2.21 | 1.18±0.50 | 7.61±0.63 | 0.26±0.02 | 1.15±0.65 |
| **S13** | 0.05±0.03 | 2.00±1.73 | 0.01±0.00 | 52.17±7.54 | 2.15±0.20 | 9.65±2.72 | 1.00±0.11 | 2.72±0.20 |
| **S14** | 0.05±0.01 | 2.00±1.73 | 0.01±0.00 | 60.49±12.39 | 8.49±4.84 | 18.37±9.11 | 1.24±0.55 | 9.64±5.68 |
| **S15** | 0.07±0.04 | 1.50±2.12 | 0.03±0.03 | 79.83±11.78 | 8.96±1.61 | 14.25±3.88 | 1.28±0.48 | 7.51±1.99 |
| **S16** | 0.07±0.01 | 2.00±1.73 | 0.03±0.03 | 62.64±3.43 | 4.43±0.61 | 11.80±0.61 | 0.73±0.57 | 4.06±0.49 |
| **S17** | 0.06±0.02 | 2.00±1.73 | 0.01±0.01 | 57.20±5.45 | 0.94±0.17 | 7.22±1.88 | 0.41±0.07 | 0.88±0.04 |
| **S18** | 0.09±0.04 | 2.00±1.73 | 0.01±0.02 | 61.24±5.25 | 1.25±0.12 | 8.86±2.40 | 0.44±0.07 | 1.34±0.10 |
| **S19** | 0.07±0.01 | 2.00±1.73 | 0.01±0.00 | 51.59±1.69 | 1.30±0.45 | 10.71±0.64 | 0.82±0.97 | 1.45±0.92 |
| **S20** | 0.03±0.01 | 2.00±1.73 | nd | 48.98±1.61 | 1.23±0.28 | 9.81±4.86 | 0.29±0.11 | 1.33±0.45 |
| **S21** | 0.07±0.02 | 2.00±1.73 | 0.01±0.01 | 49.93±0.84 | 2.40±0.40 | 8.80±1.14 | 0.56±0.05 | 2.71±0.08 |
| **S22** | 0.07±0.02 | 2.00±1.73 | 0.03±0.03 | 54.86±4.19 | 4.91±1.47 | 21.11±4.48 | 1.86±0.59 | 8.10±2.67 |
| **S23** | 0.04±0.01 | 1.50±2.12 | 0.09±0.11 | 59.37±9.57 | 3.64±2.40 | 12.52±6.24 | 0.88±0.43 | 5.58±3.83 |
| **S24** | 0.04±0.01 | 2.00±1.73 | nd | 48.72±1.50 | 1.68±0.25 | 11.85±0.92 | 0.48±0.24 | 1.90±0.16 |
| **S25** | 0.07±0.01 | 1.50±2.12 | 0.01±0.02 | 53.10±3.54 | 3.07±0.52 | 18.89±3.83 | 0.82±0.06 | 6.78±2.19 |
| **S26** | 0.10±0.00 | nd | nd | 67.60±0.00 | 4.61±0.00 | 17.10±0.00 | 0.88±0.00 | 8.79±0.00 |
| **S27** | 0.05±0.01 | 2.00±1.73 | 0.01±0.01 | 59.07±6.15 | 2.41±0.23 | 11.80±4.61 | 0.51±0.12 | 2.50±0.14 |
| **S28** | 0.06±0.02 | 1.50±2.12 | 0.02±0.00 | 60.44±8.40 | 2.84±0.80 | 7.66±3.05 | 1.00±0.74 | 3.00±1.19 |
| **S29** | 0.04±0.02 | 2.00±1.73 | 0.01±0.01 | 64.22±17.43 | 1.52±0.40 | 16.08±1.72 | 0.57±0.25 | 2.75±0.93 |
| **S30** | 0.03±0.01 | 2.00±1.73 | 0.01±0.01 | 51.94±4.96 | 1.77±0.67 | 7.47±3.07 | 0.30±0.08 | 351.95±605.39 |
| **S31** | 0.03±0.01 | 2.00±1.73 | 0.01±0.00 | 59.42±8.48 | 10.49±14.48 | 7.79±2.53 | 0.81±0.30 | 9.11±12.36 |
| **S32** | 0.05±0.00 | nd | nd | 86.30±0.00 | 3.62±0.00 | 10.20±0.00 | 0.67±0.00 | 3.85±0.00 |
| **S33** | 0.04±0.01 | 2.00±1.73 | 0.01±0.01 | 62.00±1.70 | 3.10±0.14 | 6.28±5.47 | 0.54±0.15 | 3.17±0.24 |
| **S34** | 0.04±0.00 | nd | nd | 72.40±0.00 | 4.42±0.00 | 34.10±0.00 | 0.55±0.00 | 3.98±0.00 |
| **S35** | 0.06±0.01 | 2.00±1.73 | 0.02±0.03 | 61.32±9.12 | 7.80±4.12 | 9.65±0.86 | 0.82±0.17 | 5.40±2.00 |
| **S36** | 0.06±0.03 | 2.00±1.73 | 0.07±0.07 | 71.00±10.99 | 16.58±9.79 | 13.41±5.16 | 1.22±0.58 | 11.44±6.24 |
| **S37** | 0.13±0.05 | 2.00±1.73 | 0.00±0.01 | 67.52±10.76 | 5.52±0.51 | 32.05±3.41 | 0.72±0.02 | 3.89±0.50 |
| **S38** | 0.05±0.01 | 2.00±1.73 | 0.01±0.01 | 25.79±10.16 | 3.90±1.85 | 18.07±2.98 | 0.84±0.22 | 3.31±1.28 |
| **S39** | 0.09±0.04 | 2.00±1.73 | nd | 55.57±6.78 | 17.29±3.21 | 54.62±7.41 | 0.90±0.17 | 12.25±1.68 |
| **S40** | 0.08±0.04 | 2.00±1.73 | 0.01±0.01 | 50.94±2.46 | 23.70±3.27 | 60.91±6.67 | 2.21±0.84 | 18.24±4.06 |
| **S41** | 0.08±0.02 | 2.00±1.73 | nd | 41.06±7.92 | 7.46±2.84 | 50.10±9.81 | 0.53±0.06 | 4.98±0.68 |
| **S42** | 0.07±0.00 | 2.00±1.73 | 0.01±0.01 | 46.69±1.10 | 23.22±8.67 | 35.88±3.77 | 2.43±0.83 | 19.48±8.48 |
| **S43** | 0.13±0.10 | 1.50±1.50 | 0.09±0.08 | 86.16±21.56 | 54.55±8.36 | 47.71±0.41 | 6.00±3.79 | 46.65±11.76 |
| **S44** | 0.07±0.05 | 1.50±1.50 | 0.09±0.09 | 58.93±4.17 | 32.13±5.32 | 28.25±4.15 | 2.32±0.34 | 31.68±7.18 |

Abbreviations; SO_4_: sulfate, Brm: bromide, Fe: iron, Ca: calcium, Mg: magnesium, K: potassium, Na: sodium, F: fluorine, Cl: chloride, NO_3_: nitrate, NO_2_: nitrite, NH_4_: ammonium, TOC: total organic carbon and Al: aluminum.
