# Supplementary material for: Determination of ecological statuses of streams in the Ceyhan River Basin using composition and ecological characteristics of diatoms
Source: Environ Sci Pollut Res Int. 2024 May 7;31(23):34738–55. doi: 10.1007/s11356-024-33518-0 (PMC11136811; doi:10.1007/s11356-024-33518-0)
Supplement: Supplementary file 4 — Supplementary file4 (DOCX 73 KB) [file 11356_2024_33518_MOESM4_ESM.docx]

**Supplementary 4**. The percentages of diatom species employed to calculate the scores of diatom indices aiming to assess the ecological statuses of the streams within the Ceyhan River Basin.

| **Station** | **TIT** | **TI** | **EPI-D** | **IPS** | **TDI** | **RRDI** | **DEQI** | **TWQI** | **DDI** |
| --- | --- | --- | --- | --- | --- | --- | --- | --- | --- |
| S01 | 36.0 | 51.5 | 51.9 | 74.5 | 35.9 | 19.2 | 26.5 | 16.3 | 51.0 |
| S02 | 28.6 | 48.5 | 44.6 | 71.6 | 38.6 | 17.6 | 24.8 | 14.8 | 40.3 |
| S03 | 43.1 | 56.5 | 58.3 | 68.7 | 47.8 | 20.4 | 37.9 | 14.6 | 46.0 |
| S04 | 28.2 | 52.8 | 43.5 | 66.4 | 31.2 | 16.7 | 27.1 | 19.5 | 41.2 |
| S05 | 35.3 | 58.8 | 47.1 | 88.2 | 47.1 | 23.5 | 35.3 | 17.6 | 58.8 |
| S06 | 43.8 | 62.8 | 60.5 | 73.7 | 41.2 | 26.3 | 39.1 | 27.8 | 52.9 |
| S07 | 47.2 | 51.9 | 54.3 | 79.0 | 39.0 | 22.4 | 32.6 | 18.9 | 47.6 |
| S08 | 36.2 | 52.6 | 50.3 | 79.9 | 42.1 | 20.0 | 23.9 | 12.9 | 50.1 |
| S09 | 42.0 | 50.6 | 56.6 | 71.3 | 37.0 | 18.5 | 29.5 | 18.6 | 50.5 |
| S10 | 50.0 | 65.0 | 65.0 | 85.0 | 70.0 | 15.0 | 50.0 | 20.0 | 60.0 |
| S11 | 31.2 | 49.5 | 45.7 | 70.2 | 39.4 | 18.0 | 26.9 | 17.9 | 37.4 |
| S12 | 36.8 | 60.2 | 58.4 | 68.0 | 49.5 | 21.7 | 35.7 | 28.5 | 57.1 |
| S13 | 38.4 | 54.0 | 46.8 | 68.0 | 35.0 | 18.3 | 33.5 | 18.6 | 39.5 |
| S14 | 37.6 | 61.2 | 52.2 | 79.7 | 47.2 | 16.3 | 22.5 | 19.7 | 41.6 |
| S15 | 42.1 | 58.4 | 57.2 | 73.8 | 40.9 | 21.6 | 28.7 | 18.7 | 49.3 |
| S16 | 34.2 | 52.7 | 48.1 | 72.7 | 38.9 | 21.6 | 28.9 | 16.4 | 47.0 |
| S17 | 41.3 | 56.6 | 54.4 | 68.3 | 42.5 | 18.0 | 26.6 | 14.5 | 37.6 |
| S18 | 26.4 | 52.2 | 47.4 | 59.0 | 36.8 | 17.6 | 32.7 | 16.7 | 40.1 |
| S19 | 43.6 | 59.0 | 54.9 | 70.4 | 45.7 | 20.6 | 37.3 | 21.9 | 50.4 |
| S20 | 38.0 | 55.0 | 48.5 | 71.2 | 44.0 | 18.1 | 33.5 | 15.8 | 40.6 |
| S21 | 33.0 | 42.8 | 46.9 | 60.3 | 40.0 | 15.9 | 32.2 | 14.7 | 41.2 |
| S22 | 40.8 | 66.4 | 62.9 | 77.8 | 40.6 | 18.7 | 31.2 | 24.5 | 47.6 |
| S23 | 33.8 | 50.1 | 48.2 | 70.7 | 36.7 | 18.2 | 28.6 | 14.0 | 43.2 |
| S24 | 31.2 | 50.2 | 45.8 | 64.7 | 34.5 | 15.8 | 25.5 | 16.7 | 46.8 |
| S25 | 27.0 | 51.9 | 44.7 | 73.4 | 46.8 | 13.1 | 29.1 | 13.1 | 50.6 |
| S26 | 52.4 | 57.1 | 57.1 | 66.7 | 47.6 | 9.5 | 28.6 | 23.8 | 38.1 |
| S27 | 33.8 | 55.4 | 55.3 | 73.4 | 40.1 | 18.2 | 32.9 | 12.4 | 44.4 |
| S28 | 44.7 | 59.3 | 59.3 | 77.9 | 53.9 | 16.9 | 35.5 | 24.6 | 48.4 |
| S29 | 32.0 | 48.4 | 50.7 | 69.0 | 39.4 | 16.8 | 28.6 | 15.7 | 49.4 |
| S30 | 31.1 | 55.6 | 49.6 | 70.3 | 40.1 | 18.2 | 24.7 | 13.6 | 40.8 |
| S31 | 34.7 | 50.3 | 52.6 | 66.7 | 41.2 | 17.2 | 30.9 | 14.9 | 43.9 |
| S32 | 40.0 | 50.0 | 53.3 | 80.0 | 40.0 | 20.0 | 33.3 | 23.3 | 53.3 |
| S33 | 39.0 | 56.1 | 51.9 | 70.8 | 37.5 | 21.3 | 29.5 | 19.3 | 39.8 |
| S34 | 37.5 | 62.5 | 54.2 | 87.5 | 45.8 | 12.5 | 41.7 | 25.0 | 54.2 |
| S35 | 31.7 | 40.6 | 40.9 | 58.2 | 30.1 | 10.5 | 20.8 | 12.6 | 33.7 |
| S36 | 28.4 | 47.0 | 40.3 | 62.7 | 39.1 | 17.5 | 23.3 | 16.5 | 40.2 |
| S37 | 33.7 | 48.2 | 48.3 | 68.3 | 37.0 | 20.2 | 26.8 | 22.4 | 45.8 |
| S38 | 37.8 | 61.0 | 53.4 | 66.0 | 38.5 | 18.7 | 33.2 | 23.7 | 45.8 |
| S39 | 37.4 | 56.1 | 53.8 | 75.2 | 38.7 | 16.4 | 26.9 | 20.5 | 48.7 |
| S40 | 29.1 | 53.5 | 40.4 | 63.9 | 34.8 | 20.1 | 26.3 | 16.4 | 39.0 |
| S41 | 21.0 | 41.9 | 30.6 | 59.0 | 37.7 | 13.9 | 11.6 | 15.6 | 29.5 |
| S42 | 31.3 | 49.5 | 46.2 | 62.1 | 38.3 | 13.9 | 23.6 | 16.2 | 41.8 |
| S43 | 26.7 | 42.7 | 40.4 | 67.5 | 33.2 | 15.6 | 22.1 | 11.4 | 37.7 |
| S44 | 32.9 | 58.5 | 54.2 | 65.4 | 36.4 | 25.2 | 27.5 | 15.6 | 44.2 |
