# Supplementary material for: Determination of ecological statuses of streams in the Ceyhan River Basin using composition and ecological characteristics of diatoms
Source: Environ Sci Pollut Res Int. 2024 May 7;31(23):34738–55. doi: 10.1007/s11356-024-33518-0 (PMC11136811; doi:10.1007/s11356-024-33518-0)
Supplement: Supplementary file 5 — Supplementary file5 (DOCX 48 KB) [file 11356_2024_33518_MOESM5_ESM.docx]

**Supplementary 5**. Individual (valves) numbers used in diatom indices for the biological evaluation of Ceyhan basin sampling stations based on three seasons.

| **Station** | **TIT** | **TI** | **EPI-D** | **IPS** | **TDI** | **RRDI** | **DEQI** | **TWQI** | **DDI** |
| --- | --- | --- | --- | --- | --- | --- | --- | --- | --- |
| **S01** | 206 | 372 | 233 | 601 | 220 | 86 | 186 | 99 | 327 |
| **S02** | 298 | 619 | 532 | 979 | 647 | 189 | 277 | 148 | 644 |
| **S03** | 431 | 639 | 597 | 679 | 373 | 246 | 355 | 139 | 392 |
| **S04** | 329 | 429 | 465 | 582 | 262 | 241 | 288 | 205 | 411 |
| **S06** | 456 | 615 | 577 | 866 | 506 | 261 | 357 | 264 | 631 |
| **S07** | 459 | 585 | 578 | 828 | 394 | 253 | 371 | 269 | 628 |
| **S08** | 644 | 1002 | 1012 | 1519 | 848 | 527 | 598 | 492 | 1137 |
| **S09** | 497 | 535 | 664 | 919 | 421 | 139 | 296 | 230 | 635 |
| **S11** | 293 | 313 | 298 | 564 | 267 | 65 | 181 | 172 | 328 |
| **S12** | 529 | 657 | 687 | 1204 | 772 | 291 | 465 | 292 | 635 |
| **S13** | 555 | 726 | 657 | 1152 | 480 | 270 | 448 | 261 | 537 |
| **S14** | 489 | 824 | 739 | 1188 | 780 | 251 | 468 | 324 | 807 |
| **S16** | 270 | 452 | 389 | 655 | 364 | 201 | 311 | 147 | 371 |
| **S17** | 363 | 363 | 370 | 405 | 179 | 109 | 176 | 109 | 231 |
| **S18** | 258 | 272 | 387 | 389 | 187 | 117 | 190 | 59 | 276 |
| **S19** | 469 | 600 | 496 | 762 | 360 | 156 | 241 | 192 | 511 |
| **S20** | 483 | 673 | 483 | 1000 | 446 | 237 | 364 | 276 | 444 |
| **S21** | 420 | 488 | 540 | 523 | 498 | 90 | 367 | 189 | 398 |
| **S22** | 435 | 763 | 727 | 835 | 495 | 198 | 365 | 274 | 588 |
| **S24** | 324 | 428 | 457 | 643 | 273 | 192 | 193 | 121 | 461 |
| **S27** | 476 | 773 | 809 | 1287 | 483 | 232 | 525 | 154 | 877 |
| **S28** | 687 | 1154 | 1259 | 1551 | 1099 | 607 | 862 | 397 | 1226 |
| **S29** | 259 | 462 | 430 | 547 | 346 | 121 | 231 | 173 | 406 |
| **S30** | 321 | 301 | 382 | 605 | 261 | 199 | 244 | 190 | 437 |
| **S31** | 323 | 516 | 506 | 574 | 412 | 228 | 310 | 215 | 566 |
| **S33** | 199 | 343 | 313 | 525 | 197 | 174 | 147 | 91 | 362 |
| **S35** | 477 | 397 | 427 | 693 | 285 | 129 | 196 | 111 | 372 |
| **S36** | 448 | 697 | 544 | 777 | 575 | 172 | 283 | 247 | 422 |
| **S37** | 341 | 416 | 442 | 972 | 358 | 105 | 226 | 235 | 558 |
| **S38** | 487 | 696 | 606 | 780 | 431 | 291 | 321 | 318 | 593 |
| **S39** | 281 | 370 | 398 | 634 | 226 | 134 | 223 | 166 | 458 |
| **S40** | 321 | 406 | 369 | 1140 | 252 | 121 | 297 | 114 | 502 |
| **S41** | 302 | 360 | 357 | 669 | 279 | 141 | 141 | 143 | 364 |
| **S42** | 417 | 778 | 763 | 1068 | 437 | 298 | 465 | 303 | 751 |
| **S43** | 493 | 854 | 839 | 1144 | 513 | 374 | 541 | 379 | 827 |
| **S44** | 456 | 817 | 802 | 987 | 476 | 337 | 504 | 342 | 790 |
